# Semantic and Syntactic Interference in Sentence Comprehension: A Comparison of Working Memory Models

**DOI:** 10.3389/fpsyg.2017.00198

**Published:** 2017-02-15

**Authors:** Yingying Tan, Randi C. Martin, Julie A. Van Dyke

**Affiliations:** ^1^Department of Psychology, Rice UniversityHouston, TX, USA; ^2^Haskins LaboratoriesNew Haven, CT, USA

**Keywords:** retrieval interference, working memory capacity, cue-based retrieval, sentence comprehension

## Abstract

This study investigated the nature of the underlying working memory system supporting sentence processing through examining individual differences in sensitivity to retrieval interference effects during sentence comprehension. Interference effects occur when readers incorrectly retrieve sentence constituents which are similar to those required during integrative processes. We examined interference arising from a partial match between distracting constituents and syntactic and semantic cues, and related these interference effects to performance on working memory, short-term memory (STM), vocabulary, and executive function tasks. For online sentence comprehension, as measured by self-paced reading, the magnitude of individuals' syntactic interference effects was predicted by general WM capacity and the relation remained significant when partialling out vocabulary, indicating that the effects were not due to verbal knowledge. For offline sentence comprehension, as measured by responses to comprehension questions, both general WM capacity and vocabulary knowledge interacted with semantic interference for comprehension accuracy, suggesting that both general WM capacity and the quality of semantic representations played a role in determining how well interference was resolved offline. For comprehension question reaction times, a measure of semantic STM capacity interacted with semantic but not syntactic interference. However, a measure of phonological capacity (digit span) and a general measure of resistance to response interference (Stroop effect) did not predict individuals' interference resolution abilities in either online or offline sentence comprehension. The results are discussed in relation to the multiple capacities account of working memory (e.g., Martin and Romani, 1994; Martin and He, 2004), and the cue-based retrieval parsing approach (e.g., Lewis et al., 2006; Van Dyke et al., 2014). While neither approach was fully supported, a possible means of reconciling the two approaches and directions for future research are proposed.

## Introduction

Understanding spoken or written language in real time is essential to our daily life. The ubiquitous presence of long distance linguistic dependencies (e.g., subject—verb dependencies across a relative clause as, for example, in “The *director* who embarrassed the actor *apologized*”) indicates that some type of memory representation is needed for successful integration of the dependent items. Although, there has been a long history of investigation into the role of working memory (WM) in sentence comprehension, controversy remains regarding the kind of memory system that is critical for online sentence parsing.

Most studies of the role of WM in sentence processing have focused on capacity demands involved in maintaining constituents prior to integration or maintaining predictions of upcoming syntactic structure (Daneman and Carpenter, 1980; King and Just, 1991; Just and Carpenter, 1992; Gibson, 1998, 2000; Gordon et al., 2001, 2002, 2004; Warren and Gibson, 2002; Fedorenko et al., 2006, 2007; Daneman and Hannon, 2007). For example, capacity-based accounts attribute the standard advantage in speed and accuracy for processing subject relative clauses (SRCs, as in 1a) compared to object relative clauses (ORCs, as in 1b) to increased WM demands imposed by ORC constructions (Gibson, 1998, 2000; Warren and Gibson, 2002).

(1a) SRC: The reporter who attacked the senator admitted the error.
(1b) ORC: The reporter who the senator attacked admitted the error.

Sentences like (1a) could be interpreted in a largely incremental fashion. The attachment of the gap in subject position of the RC to “*reporter*” happens immediately as the verb “*attacked*” is parsed. However, according to the WM capacity account, comprehenders have to hold “*reporter*” in sentence (1b) across some new discourse referents (e.g., the object referent “*senator*”) before attaching it to “*attacked*” as an object. Thus, the activation of “*reporter*” decays more in the ORC structure due to more discourse referents being processed and fewer resources being available for maintaining syntactic representations, as there is assumed to be a trade-off between processing and maintenance in most capacity models.

A more recent body of work has focused on interference as an explanation for these effects. These studies emphasize the content of memory representations, rather than the quantity of information that can be actively maintained in memory (Gordon et al., 2001, 2004, 2006; Van Dyke and Lewis, 2003; Lewis et al., 2006; Van Dyke and McElree, 2006, 2011; Van Dyke, 2007). For instance, Gordon et al. (2001, 2004) demonstrated that the standard disadvantage for ORCs compared to SRCs was substantially reduced when the two noun phrases (NPs) prior to the verb had dissimilar referential properties—that is, when the embedded clause NP (e.g., “*the senator*” in 1a and 1b) was replaced by a pronoun (e.g., “*you*”) or proper name (e.g., “*Joe*”). They concluded that their results favored a similarity-based interference account in which memory retrieval was hampered by similarity in the referential properties of the constituent NPs. These findings were not compatible with a pure memory capacity-based account, since the WM loads were the same across noun distinctiveness conditions. Importantly, however, Van Dyke and Lewis (2003) and Van Dyke (2007) have demonstrated that the same nouns may cause more or less interference in sentence comprehension depending on their syntactic roles in the sentence. For example, for sentences (2a and 2b) below, longer processing times at the main verb “*was complaining*” and greater comprehension errors were observed for sentences like (2b), where the noun phrase (i.e., “*warehouse*”) was a syntactic subject than when it was the object of a prepositional phrase (as in 2a), although the distances between “was complaining” and its subject “resident” were the same.

(2a) The worker was surprised that the resident who was living near the dangerous warehouse was complaining about the investigation.
(2b) The worker was surprised that the resident who said that the warehouse was dangerous was complaining about the investigation.

Van Dyke and colleagues (Van Dyke and Lewis, 2003; Van Dyke, 2007) attributed this effect to the fact that comprehenders need to retrieve the main clause subject in order to integrate it with the main verb (“was *complaining*”). The fact that “*warehouse*” is a syntactic subject in (2b) causes more interference in locating the appropriate subject (“*worker*”) than when “*warehouse*” is a prepositional object as in (2a). Relatedly, greater difficulty is observed when the intervening noun phrase has semantic properties that make it more plausible as the subject of the main clause verb. For example, longer reading times and more errors to comprehension questions are observed for sentences like (2c) than (2b), due to “*neighbor*” being a more plausible subject of “*was complaining*” than “*warehouse*.” Thus, these studies demonstrated both syntactic and semantic interference effects during sentence processing.

(2c) The worker was surprised that the resident who said that the neighbor was dangerous was complaining about the investigation.

To explain these and other interference effects, Lewis et al. have advocated a cue-based parsing approach to sentence comprehension (McElree, 2000; McElree et al., 2003; Van Dyke and Lewis, 2003; Lewis et al., 2006; Van Dyke and McElree, 2006, 2011; Van Dyke, 2007). According to this approach, sentence parsing is accomplished through a series of efficient cue-based memory retrievals. The retrieval cues are a subset of the features of the item to be retrieved, and they are derived from the incoming word, context, and grammatical knowledge. Using evidence from both empirical studies and computational modeling, these researchers have suggested that sentence processing is constrained by the degree of interference from non-target constituents, instead of storage demands. Importantly, Lewis and colleagues found that the degree of interference as predicted by the cue-based parsing theory could successfully account for effects observed in previous studies that had been attributed to storage demands, such as longer reading times (RTs) in ORC vs. SRC structures (Van Dyke and Lewis, 2003; Lewis and Vasishth, 2005; Lewis et al., 2006). In addition to the assumption of an extra retrieval in linking the object gap to the relative pronoun in ORCs, this approach suggests difficulty for ORCs due to interference from “*reporter*” while binding “*senator*” as the subject of the embedded verb in sentence 1. That is, in the ORC, when processing “*attacked*,” comprehenders need to retrieve a syntactic subject that is semantically plausible as its agent (Van Dyke, 2007). “*Reporter*” fits both of these requirements. In contrast, in the SRC, there is no other preceding noun phrase to provide interference.

## Individual differences in WM and their role in sentence processing

As the cue-based retrieval approach focuses on interference as the source of difficulty in sentence processing, there is less need to resort to memory capacity as an explanatory factor *per se*. The move away from capacity accounts is consistent with recent embedded processes accounts of WM, which define WM as the activated portion of long-term memory, together with a very small number of these activated items (e.g., from one to four) within the focus of attention (Lewis, 1996; Cowan, 1999, 2000; Oberauer, 2002; McElree, 2006; Öztekin et al., 2010). In the embedded processes view, information outside the focus is accessed via cue-based retrieval. Consonant with this WM approach, the cue-based parsing model of sentence processing assumes that skilled sentence processing depends on the maintenance of as few as 1–2 chunks of information in WM. Information outside the focus is retrieved through a match of retrieval cues to stored representations. These hypotheses have been supported through a computational implementation (Lewis and Vasishth, 2005; Lewis et al., 2006), and behavioral studies, using a range of methodologies, including self-paced reading, eye-tracking, and speed-accuracy tradeoff techniques (McElree and Dosher, 1989; McElree, 2000, 2006; Van Dyke and Lewis, 2003; Van Dyke and McElree, 2006, 2011; Van Dyke, 2007; Van Dyke and Johns, 2012). The claims from the cue-based parsing approach challenge research pointing to individual differences in WM capacity as a source of sentence processing difficulty (Daneman and Carpenter, 1980; Fedorenko et al., 2006, 2007; Daneman and Hannon, 2007; Unsworth and Engle, 2007). If skilled sentence processing requires at most 1–2 chunks of active memory, this renders moot the claim that poor sentence comprehension is due to low WM capacity.

However, despite the substantial evidence in favor of such a highly limited WM capacity, some criticisms have been raised (Cowan, 2011; Caplan and Waters, 2013). Even among memory researchers who endorse an embedded processes account of WM, there is an ongoing debate about the storage limits of the focus of attention. For example, while McElree and colleagues (e.g., McElree and Dosher, 1989; Öztekin et al., 2010) have estimated the capacity of the focus of attention to be only one chunk of information based on behavioral and neuroimaging results, Cowan (2011) has critiqued these claims and argued for a multi-item focus of attention with a limit of about 3 or 4 chunks. With a larger capacity for the focus of attention, the possibility remains of meaningful individual differences in this capacity. In fact, Unsworth et al. (2014) and Shipstead et al. (2014) have argued, based on a confirmatory factor analytic approach, that a contributing factor to variation in working memory capacity is the capacity of the focus of attention. Therefore, it remains important to examine whether measures of working memory capacity predict comprehension performance, once other relevant factors have been controlled for.

Historically, a substantial body of research has shown a relation between WM capacity measures and the ability to process complex sentences (Daneman and Carpenter, 1980; King and Just, 1991; Just and Carpenter, 1992; Gibson, 1998; Fedorenko et al., 2006, 2007; Daneman and Hannon, 2007; see Long et al., 2006, for a review). Most commonly, these studies have used complex span measures, such as reading span and operation span to index WM capacity (Daneman and Carpenter, 1980; Turner and Engle, 1989). These measures involve both processing and storage components, in that individuals carry out some processing task (e.g., sentence verification in the reading span task or arithmetic computations in the operation span task) while simultaneously maintaining a secondary verbal load (e.g., words or letters). The claim has thus been that these measures reflect a single capacity that can be flexibly allocated to either processing or storage (Just and Carpenter, 1992). In the sentence comprehension domain, storage could involve maintenance of, for instance, lexical items or conceptual representations, and processing could involve, for instance, accessing these representations or assigning thematic roles.

The importance of using WM measures that combine both processing and storage has been emphasized, as other indices of WM capacity such as standard digit or word span, which mainly reflect storage of phonological representations (Baddeley et al., 1998) have typically shown little relation with the ability to process syntactically complex sentences (Waters et al., 1991; Martin and Romani, 1994; Daneman and Merikle, 1996; Caplan and Waters, 1999; Hanten and Martin, 2000; Friedmann and Gvion, 2003; Daneman and Hannon, 2007; Caplan et al., 2013; Kush et al., 2015). Some recent studies, however, have provided some support for a role for phonological storage in complex sentence comprehension (Acheson and MacDonald, 2011; Pettigrew and Hillis, 2014), suggesting that the issue may warrant further attention.

Even though complex span measures have more consistently shown a relation to sentence processing, the source of the WM-language relationship remains unclear, as WM capacity might relate to various aspects of comprehension. For example, Caplan and Waters (1999) showed that many of the findings relating complex span measures to *online* comprehension ability failed to replicate or did not support the conclusions that had been drawn. In their own work, they found that neither simple span (e.g., digit span) nor complex span measures related to *online* measures of syntactic processing ability—that is, measures related to the processing of each word as the sentence unfolds. However, these measures did relate to *offline* processing ability, which involves using the products of comprehension to carry out some task such as matching a sentence to a picture. The implication is that the WM tapped by span tasks is involved in reviewing or checking the results of comprehension rather than the initial interpretation of a sentence. Thus, Caplan and Waters (1999) concluded that for online sentence processing, a WM system specialized for language interpretation is involved. More recent work by Caplan and colleagues (Caplan et al., 2013; Evans et al., 2015) has supported these conclusions.

In contrast to the inconsistent results for complex span measures and simple span measures tapping phonological storage, one measure which has been consistently related to argument integration during sentence processing is the category probe task, an index of semantic short-term memory (STM) in which participants are presented with a word list and asked to judge whether a probe word is in the same semantic category as any list word (Martin and Romani, 1994; Martin et al., 1994; Hanten and Martin, 2000; Martin and He, 2004; Martin, 2005; Harris et al., 2013). Martin et al. (1994); Martin and He (2004) reported a double dissociation between aphasic patients with semantic STM deficits and patients with phonological STM deficits, with the two types of STM deficits having different consequences for sentence comprehension. Aphasic patients with impaired semantic STM but relatively spared phonological retention had difficulty in understanding sentences in which the integration of semantic information of words was delayed rather than immediate. For example, when detecting the anomaly in sentences in which one to three nouns appeared before a verb (e.g., “Rugs cracked during the move”; “*Rugs, vases, and mirrors cracked during the move*”) relative to sentences in which the nouns followed the verb (e.g., “The movers cracked the rugs”; “*The movers cracked the mirrors, vases, and rugs*”), performance was equivalent and at a high level when there was only one noun preceding or following the verb, but declined substantially with increasing numbers of nouns before the verb, but remained at the same high level with increasing numbers of nouns after the verb. Similar results were obtained for sentences with varying numbers of adjectives before a noun (“The rusty old red swimsuit”…) vs. after a noun (e.g., “The swimsuit was old, red, and rusty…). In contrast, patients with a phonological STM deficit showed a normal pattern of effects of the delayed vs. immediate integration conditions but had difficulty with sentence repetition (Hanten and Martin, 2000; Martin and He, 2004). Interestingly, the patients with semantic STM deficits performed at a high level and showed no effect of distance on a grammaticality judgment task that varied the distance between words signaling a grammatical error in ten different types of sentence structures (e.g., for verb phrase deletion: “The hopeful young contestants didn't win and neither ^*^was their rather aggressive competitor” vs. “Susan didn't leave despite many hints from her tired hosts and neither ^*^was Mary”). In contrast, one patient who had little deficit in either semantic or phonological STM demonstrated a detrimental effect of distance in this grammaticality judgment task (Martin and Romani, 1994; Martin and He, 2004). Thus, Martin and colleagues put forward a multiple-component model within the language processing domain, with separate capacities for the retention of phonological, semantic, and syntactic information (Martin and Romani, 1994; Martin and Saffran, 1997; Martin and Freedman, 2001; Martin et al., 2003; Martin and He, 2004; Hamilton et al., 2009). According to this model, semantic and syntactic STM capacities, but not phonological STM, are critical for maintaining unintegrated word meanings and syntactic structures during sentence comprehension, respectively.

However, as discussed earlier, the cue-based parsing approach challenges the long-standing assumption that individual differences in WM capacity are a source of the variation in sentence processing ability, and provides an alternative explanation of the prior neuropsychological results. According to the cue-based parsing approach, comprehension difficulty may arise either from variation in the quality of to-be-retrieved representations, or variation in the ability to efficiently use retrieval cues to activate target information and inhibit irrelevant information (Van Dyke et al., 2014). These two accounts point to language experience and executive control ability as playing important roles for determining comprehension ability. Thus, the relation between semantic STM and sentence comprehension might actually reflect underlying deficits in semantic knowledge representations, which resulted in less rich encoding of semantic features during sentence processing. This assumption is partially supported by the finding that even though the patients with semantic STM deficits in Martin et al.'s studies performed well in terms of accuracy on single word semantic tasks, they did show some deficits in latencies on certain timed semantic tasks (Martin and Romani, 1994; Martin and He, 2004). Thus, these patients may have had some mild degree of semantic deficit *per se* that affected their comprehension. Another possibility is that these patients have a deficit in the mechanism employed for interference resolution. There are findings suggesting that the left inferior frontal region damaged in the patients with semantic STM deficits is crucial for aspects of executive function (Hamilton and Martin, 2005, 2007), i.e., “semantic control” according to Lambon Ralph and colleagues (Jefferies et al., 2007; Whitney et al., 2011) and “selection from competitors” according to Thompson-Schill and colleagues (Novick et al., 2009; Barde et al., 2010), both of which might be involved in interference resolution during sentence comprehension. Thus, to evaluate the multiple capacities hypothesis, it would be important to show that semantic capacity predicts semantic interference resolution, even after taking into account variations in semantic knowledge and executive control.

Thus, far there has been only a single study to investigate individual differences in sensitivity to interference during sentence processing which takes into account variation in language knowledge. Van Dyke et al. (2014) utilized a dual-task paradigm to assess participants' ability to suppress proactive interference from distractors that appeared in a 3-word memory list (e.g., TABLE, SINK, TRUCK) prior to reading the critical sentence. The critical contrast was between the sentences where the verb was manipulated as follows: *It was boat that the guy who drank coffee FIXED/SAILED for 2 sunny days*. A previous study (Van Dyke and McElree, 2006) with university-level participants demonstrated that when the verb appeared as *fixed*, participants experienced retrieval interference from the items in the memory list (which are all fixable items). The Van Dyke et al. (2014) study sampled from a broader range of ability levels and administered a comprehensive battery of 24 individual differences measures. After partialling out the variance that each measure shared with a composite measure of IQ (combining the vocabulary and matrix reasoning subtests of the Weschler Abbreviated Scale of Intelligence; Psychological Corp.; Wechsler, 1994/1999), they observed that WM capacity no longer interacted with individuals' sensitivity to interference whereas a receptive vocabulary measure (Peabody Picture Vocabulary Test-Revised; Dunn and Dunn, 1997) did, such that the comprehension for individuals with low vocabulary was more affected by interference. Van Dyke et al. interpreted this result as most consistent with the view that the quality of to-be-retrieved representations (assumed to be reflected in the receptive vocabulary measure) is a critical determinant of sensitivity to interference. In addition, Van Dyke et al. also observed a significant interaction of IQ with sensitivity to interference, which mirrored the effect found with vocabulary: individuals with lower IQ were more affected by interference. This interaction with IQ is difficult to fully interpret in light of the findings suggesting that IQ shares significant variance with WMC and that this shared variance is itself multi-faceted (Engle et al., 1999a,b; Kane and Engle, 2002; Hambrick et al., 2005; Kane et al., 2007; Shipstead et al., 2014; Harrison et al., 2015). Due to the collinearity of fluid intelligence and WM, we included the WAIS vocabulary measure (Wechsler, 1997; WAIS-III, 2002) as a control variable, because this task is generally viewed as a measure of crystallized intelligence, which has less shared variance with WM capacity (Kane et al., 2007). The inclusion of this task provides a means of assessing the role of WM capacity independent of lexical processing ability.

For the executive control hypothesis, several prior studies have supported a role for general executive control (e.g., as measured by the verbal Stroop task) in comprehending garden-path sentences (Novick et al., 2005, 2014; Vuong and Martin, 2014; Hsu and Novick, 2016). Nevertheless, a potential problem with these findings is that the use of garden path constructions, where correct comprehension requires overriding preferred interpretations of words or syntactic structures, may engage resolution processes differently than in unambiguous sentences (i.e., they may be consciously engaged.) Thus, an important contribution of the current study is to provide data on how executive function becomes involved when parsing unambiguous sentences more like those routinely encountered in everyday conversation.

## Current study

The current study is the first to examine individual differences in resolving syntactic and semantic interference from distractors embedded within a sentence during online processing. Motivated by the studies summarized above, we examined whether interference resolution depends on general WM capacity, STM capacity (phonological or semantic), executive control abilities, and/or aspects of representational quality. Given the ongoing debates about the nature of the WM-sentence comprehension relation, we aimed to test specific hypotheses about the relation between these various tasks and language processing as predicted by different theories. We summarize these predictions in Table 1 with reference to the specific tasks we use to represent each cognitive construct (see Section Methods for task descriptions).

**Table 1:** **Predictions of the relations between individual differences measurements and interference effects**.

**Account**	**Predictions**
General WM approach (e.g., Just and Carpenter, 1992; Fedorenko et al., 2006, 2007)	Complex span measures (e.g., reading span and operation span) should correlate with the size of both semantic and syntactic interference effects.
Multiple capacities approach (e.g., Martin et al., 1999; Martin and He, 2004)	Semantic STM (e.g. category probe) should correlate with semantic interference resolution, even after controlling for verbal knowledge and executive function abilities.
	Phonological STM (e.g., digit span) should not correlate with either type of interference.
	Syntactic interference resolution ability should not correlate with either semantic STM or phonological STM^a^.
Language-specific WM approach (e.g., Caplan and Waters, 1999, 2013)	There should be no correlations between any WM span tasks and online interference effects, but only with offline interference effects.
Cue-based parsing approach (e.g., Van Dyke et al., 2014)	Representational quality (e.g., vocabulary) and/or executive function (e.g., Stroop task) should correlate with the size of interference effects.
	Additional interactions between semantic interference and WM measures may occur^b^.

a *A specific link between syntactic STM capacity and syntactic interference resolution, but not semantic resolution, should also be expected. However, at present there is no appropriate measurement for syntactic STM. Thus, the predictions from the multiple capacities approach focus on the relation between semantic interference and semantic STM capacity*.

b *The results of Van Dyke et al. (2014) suggest that these interactions should stem from variance shared between WM and IQ*.

While the above accounts entail a range of predictions, some differences between them are critical for the present study. The general WM account implies that WM measures will be related to both semantic and syntactic interference, potentially both in online and offline measures. Thus, numerous interactions between WM and sentence effects are predicted. In contrast, the multiple capacities approach predicts that only specific relations will be obtained—for instance, between a measure of semantic capacity and semantic interference resolution but not syntactic interference resolution. Thus, fewer interactions are predicted which follow specific patterns. The language-specific WM approach predicts no relations between WM measures and sentence processing measures, at least in online processing. The cue-based parsing approach predicts that language knowledge and executive function should interact with interference effects. Interactions with capacity measures are generally not expected. However, based on the findings of Van Dyke et al. (2014)—assuming they hold for cases where distractors are embedded *within* the sentence—interactions with the portion of WM capacity variance related to IQ may occur. This could be predicted for semantic interference, which is the only type of interference examined in the Van Dyke et al. study.

## Methods

### Subjects

One hundred and twenty undergraduate students (79 females) from Rice University were recruited for this experiment. Each subject participated in two 1.5 h sessions. All subjects were native English speakers without a diagnosed reading or learning disability and normal or corrected-to-normal vision. Informed consent was obtained from each subject in accordance with the guidelines and approval of the Rice University Institutional Review Board. Subjects received credit toward experiment participation requirements for their courses. Eight subjects were excluded from the analysis due to low accuracy in the sentence comprehension task (< 75%).

### Materials and procedure

#### Sentence comprehension task

##### Materials

We used modified versions of the sentences in Van Dyke's (2007) study. There were eighty sets of sentences with four different types of sentences in each set crossing two levels of syntactic interference with two levels of semantic interference (see examples in Table 2 or see Appendix A in Supplementary Material for complete list.) To increase readability, we refer to the low and high syntactic interference conditions as LoSyn and HiSyn, respectively, while the low and high semantic interference conditions are referred to as LoSem and HiSem. The four sentences in a set began with the same introduction region and differed in the intervening region, in which semantic and syntactic interference were manipulated. To avoid potential problems associated with local coherence effects (Tabor et al., 2004), an adverbial phrase was inserted after the intervening region in order to increase the separation between the interfering noun and the main clause verb. Difficulty with local coherence might have arisen particularly in the low syntactic interference conditions as the interfering NP would have appeared immediately before the main clause verb without the adverbial phrase. The main verb for the long-distance dependency was identified as the critical region, as this is the point at which comprehenders would attempt to retrieve the subject NP. The phrase following the main verb is termed the spillover region, because it is often the case that effects in one region spill over to the next region in self-paced reading (Just et al., 1982).

**Table 2:** **Example syntactic and semantic interference stimuli for experiment showing phrasal regions for self-paced reading**.

**Sentence region**		**Example stimulus**
Introduction		The critic
Intervening region	LoSyn/LoSem	Who had enjoyed the memorable play
	LoSyn/HiSem	Who had enjoyed the memorable actress
	HiSyn/LoSem	Who mentioned that the play was memorable
	HiSyn/HiSem	Who mentioned that the actress was memorable
Adverbial phrase		At the new theatre
Critical region		Will visit
Spillover region		The director

“*Lo-“ and “Hi-“ refer to low and high interference condition, while “-Syn” and “-Sem” refer to syntactic interference and semantic interference condition*.

Eighty sets of four sentences were constructed containing the four types of intervening clauses (see Appendix A in Supplementary Material). The mean length of the experimental sentences was 15.7 words (SD = 1.3 words). To avoid repetition of the verbs and sentence content within one subject, the four items in each set were assigned to four lists and each subject received only one list containing one item per set in a list. Two pseudo-randomized sequences were created for each stimulus list, resulting in a total of eight lists. Each subject saw 20 target sentences in each of the four conditions. Additionally, 80 filler items were added to each list consisting of 36 sentences with a relative clause structure (16 with ORC) and 44 non-RC sentences with right-branching structures. The ORC sentences were included in order to distract subjects from detecting the target sentences. In all, each subject saw 160 sentences during the experiment.

##### Procedure

Stimuli were presented in a phrase-by-phrase, non-cumulative, self-paced fashion (Just et al., 1982). Ten practice sentences were presented prior to the experimental sentences, consisting of 4 sentences in the same format as experimental sentences and 6 fillers. Participants were instructed to read each sentence for comprehension silently at a natural pace and told that there would be a comprehension question after each sentence. All trials began with a fixation point appearing in the center of the screen for 1,000 ms, followed by the first phrase. Participants pressed a button with their index finger to bring up the phrases in each sentence, and a period was presented together with the last phrase. The reading time (RT) was recorded as the time between stimulus onset and button press for each phrase. After each sentence, a comprehension question was presented. For the experimental sentences, the phrase probed the critical subject-verb integration (e.g., for the example sentences, “*Who will visit*?”). For the filler sentences, the comprehension questions probed other noun phrases in the sentence (e.g., for the filler sentence “*The artist who feared that the publicist would cancel the exhibit quit on his own*,” the comprehension question was “*What might be canceled?*”). Subjects were required to provide a spoken response, and speed for answering the question was measured through a voice key trigger as the time between question onset and the time when subjects start producing vocal response. The next sentence started after an inter-trial interval of 1,000 ms.

#### Working memory tasks

To tap subjects' memory capacity, we included two simple span measures and two complex span measures.

#### Simple span measures

##### Category probe task

The category probe task (Martin et al., 1994) was included to tap subjects' semantic STM. In this task, subjects were presented with an auditory word list. After a short pause, they heard a probe word and had to judge whether this word was in the same category as any of the words in the list (all of the words in a list were drawn from different categories). Before testing, subjects were shown a list of all nine categories (e.g., animals, clothing, fruits, etc.) that would be presented in the experiment as well as all the words belonging to each category. The number of words in each list ranged from 4 to 7 and there were 24 lists at each list length. The dependent measure was overall accuracy for each subject.

##### Digit span task

The digit span task from Wechsler Adult Intelligence Scale-third edition (Wechsler, 1997) was included to tap subjects' phonological STM. Participants heard a list of digits and they were required to repeat the numbers aloud in order at the end of each list. The number of digits in each list ranged from 3 to 9, and there were 2 trials at each level. Each subject completed all 14 trials. Overall accuracy was calculated for each subject.

#### Complex span measures

##### Operation span task

The automated version of Operation Span (Unsworth et al., 2005) was used to measure WM capacity. Subjects were instructed to solve a math operation [e.g., (1 ^*^ 2) + 1 = ?] as quickly as possible and then remember a single letter. During this task, a math operation was presented on the screen first. After subjects solved it and clicked the mouse, a digit appeared and subjects judged whether it was the correct answer. After a mouse click response, a letter to be recalled was shown on the screen for 800 ms. This to-be-remembered letter was followed by either another math operation-letter combination or the recall screen, which showed up at the end of each set of operation-letter pairs. At recall, subjects clicked a box next to the appropriate letters in the current set in the order presented. The experimental trials contain three trials at each set size, with set sizes ranging from 3 to 7 items, resulting in a total of 75 trials. The order of set sizes was random for each participant. We evaluated subjects' operation span by the total number of letters recalled in the correct serial position regardless of whether the entire trial was recalled correctly.

##### Reading span task

We used the automated version of reading span (Unsworth et al., 2005) modified from Daneman and Carpenter's (1980) original version. The task is very similar to the *operation span task*, but instead of solving math operations, subjects are instructed to judge whether a presented sentence makes sense or not (e.g., *Andy was stopped by the policeman because he crossed the yellow heaven*.). After pushing a button to indicate whether the sentence makes sense, a to-be-remembered letter is shown on the screen for 800 ms, which is followed by either another sentence-letter combination or the final recall screen. Set sizes ranged from 3 to 7 items. At the end of each set of sentences, subjects recalled all the letters in the current set in order by clicking boxes next to letters. This results in a total of 75 trials; the order of set sizes was random for each participant. We evaluated subjects' performance with the same scoring procedure as operation span.

#### Executive function measure

The standard verbal Stroop task (Stroop, 1935) was adopted in the current experiment to measure subjects' resistance to interference. Subjects were required to name the ink color in all conditions. In the congruent condition, a color word appeared in the congruent color (e.g., the word “blue” in blue ink) while in the incongruent condition, a color word was presented in a different ink color (e.g., the word “blue” in red ink). In the neutral condition, a series of colored asterisks was presented. There were 65 incongruent trials, 77 neutral trials, and 12 congruent trials. Response naming latencies were recorded from the onset of the stimulus through a voice key response. The Stroop interference score for each subject was calculated by subtracting the mean correct RT in the neutral condition from that in the incongruent condition.

#### Verbal knowledge measure

The vocabulary subtest of the WAIS-III (Wechsler, 1997) was administered as a measure of verbal knowledge. The test requires subjects to provide word definitions (e.g., Tell me what *confide* means). We began the vocabulary test from the 12th item in this subtest because the words before the 12th were not discriminating enough for our undergraduate students. Twenty-two words were presented. The test was scored based on the standard scoring criteria in the WAIS-III manual. Each definition received either 0, 1, or 2 points.

### General procedure

Testing was carried out in two sessions, each lasting ~1.5 h for a total of 3 h. A button box with millisecond accuracy was used for the computerized tasks and a voice key was attached to the button box to record verbal responses. The sentence reading, category probe, digit span, and Stroop tasks were conducted on a Macintosh with PsyScope (Cohen et al., 1993). The reading span and operation span tasks were run using E-Prime (Schneider et al., 2002). The task administration order was fixed for all participants: sentence comprehension task first, followed by digit span, Stroop, vocabulary, category probe, reading span, and operation span.

### Data analysis

The sentence comprehension experiment produced four dependent variables: reading times (RT) from self-paced reading in the critical region (main verb) and spillover region (the phrase following the main verb), and speed and accuracy for answering comprehension questions. For all RT analyses (i.e., self-paced reading and question answering speed), only data from accurate trials were included. Outliers were calculated by condition for each subject and reading times >2.5 standard deviations away from the mean for each condition were removed from the analyses. The trimming removed 4% of the data in the critical region, 4% of the data in the spillover region, and 5% of the data in the question answering times.

Because some researchers have claimed that variations in processing speed account for the correlations between WM capacity and performance on complex cognitive tasks (Fry and Hale, 1996; Salthouse et al., 2003), a logarithmic transformation was performed on the RT data in order to remove the effects of speed on the size of effects (Verhaeghen and De Meersman, 1998). This transformation also yields more normally distributed RTs than raw RTs, and thus the transformed data better meet the assumptions underlying the general linear model (Baayen and Milin, 2010)^1^.

The log-transformed RT and error rate data were modeled in linear mixed-effects models (LMEMs) using R (2.11.1) implemented within the *lme4* package, with a logistic linking function for dichotomous variables such as comprehension error rate (Baayen, 2008, 2011; Baayen et al., 2008) following guidelines set out by Baayen (2008). Each of the independent variables was mean-centered prior to analysis. This centering allows us to interpret results by making effects analogous to ANOVA results. The semantic and syntactic interference were coded with the low interference condition as −1 and high interference condition as 1. Thus, negative coefficients for each main effect of log RT or error proportion signify worse performance (i.e., longer RT) in the high interference conditions.

In the mixed-effects models, fixed effects included the main effects and interaction of semantic and syntactic interference manipulations, as well as the main effects of all the individual differences and their interactions with semantic/syntactic interference. In addition to these fixed effects, all the mixed-effects models included maximal random-effects structures to provide the most conservative assessment of the significance of results (Barr et al., 2013). Thus, by-subject adjustments to the intercept as well as by-subject adjustments to the random slope of interaction between semantic × syntactic interference were included in the models. Similarly, by-item adjustments to the intercept and to the random slope of interaction between semantic × syntactic interference were included. In addition, word length was included as a control factor in all the models for the critical and spillover regions. There was no convergence problem for any of the models reported in this study. Throughout, we present coefficient estimates, standard errors (SE), and *t*- or *z*-scores (when the dependent measures is a dichotomous variable, i.e., accuracy) derived from 50,000 Monte Carlo Markov Chain (MCMC) runs. For the RT data, the degrees of freedom are not reported because they can only be approximated in LMEMs, and consequently *p*-values are not reported. The *t*- or *z*-score based on MCMC sampling and *t*- or *z*-score based on the upper bound of the degrees of freedom tend to be very close for a relatively large sample (Baayen et al., 2008). Hence, we adopted a standard in which an absolute *t*- equal to or >2.0 was considered to be significant at the α = 0.05 level. For the mixed logistic regression analysis of errors, degrees of freedom can be calculated and thus, *p*-values are reported in the results for these analyses.

## Results

### Relations among individual differences measures

Range, mean, and standard deviation for each individual differences measure are shown in Table 3. Reliabilities are also reported as the extent of relation between two variables is limited by the reliability of the measures involved (Schmitt, 1996). For most measures, internal reliability was calculated as the split-half correlation adjusted with the Spearman–Brown prophecy formula (Cronbach, 1951). For operation span and reading span, the internal reliability was obtained from previous studies (Redick et al., 2012). Although, most subjects tended to perform well in most tasks, their scores were distributed widely on each scale and the reliability of all these tests was very high.

**Table 3:** **Descriptive data and reliability estimates for all the individual differences measurements**.

**Individual differences measures**	**Index**	**Mean**	**Range**	**SD**	**Skew**	**Kurtosis**	**Reliability**
Operation span	Partial score	63/75	36–75	8.5	−0.97	0.86	0.84^a^
Reading span	Partial score	62/75	26–75	10.4	−0.85	0.40	0.86^a^
Category probe	Accuracy	0.81	0.64–0.94	0.07	−0.24	−0.66	0.74^b^
Digit span	Accuracy	0.74	0.43–1.00	0.14	−0.02	−0.89	0.73^b^
Stroop^c^	RT (ms)	113	13–277	58.9	0.66	−0.10	0.89^b^
Vocabulary	Score	36/44	19–44	5.5	−0.91	0.46	0.82^b^

a *Cronbach's Alpha*.

b *Odd-even split-half reliability*.

c *Stroop effect is calculated by subtracting participants' mean RT in the neutral conditions from that in the incongruent condition*.

The correlations among the individual differences measures are shown in Table 4. Reading span and operation span had a moderately high correlation (*r* = 0.54), which is consistent with previous studies (as reported by Redick et al., 2012, mean *r* = 0.64). The category probe measure had low but significant correlations with reading span, operation span and vocabulary. The correlation between digit span and category probe was very low (*r* = 0.11), substantiating the claim that these measures tap different aspects of STM. Digit span was correlated significantly with reading span and operation span, which is consistent with other evidence showing a phonological component to these WM measures (Kane and Engle, 2003; Camos et al., 2011, 2013). In addition, Stroop correlated with both reading span and operation span, also in line with previous findings (Kane et al., 2001), but did not correlate with digit span or category probe. This pattern may be attributed to the attentional control component (i.e., interference resolution ability in the Stroop task), which is more prominent in complex span measures than in simple span measures (Engle, 2002; Kane and Engle, 2003; Engle and Kane, 2004; Unsworth et al., 2009). Lastly, vocabulary had low to moderate correlations with all of the measures except digit span and Stroop.

**Table 4:** **Full correlation matrix of the correlation tests between individual differences measures**.

**Individual differences Measures**	**Reading span**	**Operation span**	**WM composite**	**Category probe**	**Digit span**	**Stroop**
Reading span						
Operation span	0.54^**^					
WM composite	0.88^**^	0.88^**^				
Category probe	0.33^**^	0.20^*^	0.30^**^			
Digit span	0.22^*^	0.31^**^	0.31^**^	0.11		
Stroop	−0.24^*^	−0.27^**^	−0.29^**^	−0.03	−0.13	
Vocabulary	0.35^**^	0.23^*^	0.33^**^	0.21^*^	0.17	−0.17

* p < 0.05;

** *p < 0.01*.

*The WM composite variable for WM capacity was calculated by combining z-scores for reading span and operation span*.

### Semantic and syntactic interference and their relation to individual differences measures

Mean error rates and response times for comprehension questions and mean self-paced reading times for the main verb (i.e., critical region) and the following phrase (i.e., spillover region) are shown in Table 5. Subjects generally performed well on the comprehension questions (overall accuracy = 87%). The reliability of each sentence comprehension dependent measure was calculated as a split-half correlation adjusted with the Spearman–Brown prophecy formula (Cronbach, 1951). The reliability of all the dependent measures was very high (≥0.78; see Appendix C in Supplementary Material). As expected, subjects showed the lowest error rate (8%) and shortest question answering time (mean = 1,265 ms) in the LoSyn/LoSem interference condition, and the highest error rate (18%) and longest answering time (mean = 1,436 ms) in the HiSyn/HiSem interference condition.

**Table 5:** **Descriptive data of mean proportion errors and reaction time (ms) in sentence comprehension task and self-paced reading time (ms)**.

**Conditions**	**Comprehension questions**		**Self-paced reading (ms)**	
	**Error proportion**	**Speed (ms)**	**Critical region**	**Spillover region**
LoSyn/LoSem	0.08	1,265	920	957
LoSyn/HiSem	0.13	1,385	935	992
HiSyn/LoSem	0.12	1,281	913	980
HiSyn/HiSem	0.18	1,436	938	1,039

LoSyn and HiSyn refer to low and high syntactic interference conditions. LoSem and HiSem refer to low and high semantic interference conditions

To obtain a more reliable and robust measure for general WM capacity and to avoid the collinearity issue between reading span and operation span, we computed a composite WM measure by averaging *z*-scores for the two complex span measures, resulting in a measure which would increase measurement precision of the overlapping component (Nunnally et al., 1967). In order to examine the unique contribution of general WM, specific STM, or executive function as measured by each cognitive ability task, all the individual differences measures were included in the mixed-effects models simultaneously. That is, to determine whether span measures contributed to the prediction of performance beyond what could be predicted on the basis of verbal knowledge, the main effect of vocabulary and its interactions with semantic or syntactic interference as fixed effects were included in all the models with other individual difference measures. Because of the potential concern about mild multicollinearity among the individual differences measures, we also report in Appendix B in Supplementary Material the output of mixed-effects models with each individual differences measure alone (with vocabulary as a control variable)^2^. Generally, the single predictor analyses provided convergent results to the multiple predictor analyses. Thus, we will only focus on the results from multiple predictor analyses, which revealed the unique contribution of each predictor when controlling for the others.

For these analyses, we focused on the interaction between semantic/syntactic interference and the individual differences measures. In general, individuals with higher capacities or better interference resolution ability should show less difference between high vs. low interference conditions relative to subjects with lower capacities or poorer interference resolution ability. These effects should show up as significant interaction terms (i.e., interference manipulation × individual difference measure) in the mixed effects models.

#### Self-paced reading times

##### Model 1 (online measures)

Semantic/syntactic interference effects and main effects of each individual differences measure are shown in Table 6, and the interactions between sentence processing and individual differences measures are shown in Table 7. Both semantic interference (*t* = 2.56) and syntactic interference (*t* = 2.03) effects were significant in the spillover region, whereas neither was significant at the critical verb (semantic: *t* = 1.94; syntactic: *t* = −0.02). The interaction between semantic and syntactic interference was not close to significance in either region. The time course of these effects is different from that observed in Van Dyke (2007), in which the syntactic interference effect was obtained at the critical verb, whereas the semantic interference effect was only observed in the final region (after a spillover region). The discrepancies between the results of the current study and Van Dyke's (2007) study may be explained by methodological differences. For one, the current study utilized a self-paced reading paradigm, while the Van Dyke (2007) study provided eye-tracking data. As effects in self-paced reading often spill over into regions following the critical manipulation (e.g., Just and Carpenter, 1992; Bartek et al., 2011), it is possible that an earlier occurring syntactic interference effect may have only become evident in the spillover region. On the other hand, a much larger sample size was used here, which may have made provided the power to detect semantic effects earlier.

**Table 6:** **Results of semantic/syntactic interference effects and main effects of each individual differences (IDs) measure in mixed-effects analyses, in which all individual measures were included**.

	**Self-paced reading (ms)**						**Comprehension question**					
	**Critical region (RT)**			**Spillover region (RT)**			**Error proportion**			**Speed (RT)**		
	**Coefficient**	**SE**	***t-*****score**	**Coefficient**	**SE**	***t-*****score**	**Coefficient**	**SE**	***z*****-score (*p*-value)**	**Coefficient**	**SE**	***t-*****score**
Intercept	2.82800	0.02081	135.90^*^	2.86200	0.01735	164.97^*^	−1.70500	0.41310	−4.13^*^ (< 0.001)	3.07400	0.01656	185.65^*^
Length	0.00720	0.00139	5.19^*^	0.00617	0.00081	7.64^*^	−0.03351	0.01872	−1.79(0.074)	0.00130	0.00067	1.94
Semantic interf	0.00520	0.00268	1.94	0.00661	0.00259	2.56^*^	0.27850	0.04903	5.68^*^ (< 0.001)	0.01933	0.00203	9.52^*^
Syntactic interf	−0.00006	0.00286	−0.02	0.00496	0.00245	2.03^*^	0.13100	0.04764	2.75^*^(0.006)	0.00434	0.00178	2.44^*^
Sem × Syn	0.00183	0.00255	0.72	0.00119	0.00222	0.54	−0.00141	0.04641	−0.03(0.976)	0.00252	0.00179	1.41
*IDs*												
Category probe	0.61350	0.18750	3.27^*^	0.42250	0.19410	2.18^*^	−2.10900	0.83330	−2.53^*^ (0.011)	−0.01326	0.10660	−0.12
WM Composite	−0.01053	0.00860	−1.22	-0.01146	0.00890	−1.29	−0.06856	0.03766	−1.82(0.069)	−0.00238	0.00488	−0.49
Digit span	−0.02536	0.09852	−0.24	-0.06086	0.10190	−0.60	0.00962	0.44480	−0.02(0.983)	−0.08397	0.05594	−1.5
Stroop	−0.00002	0.00023	−0.07	0.00021	0.00024	0.90	0.00050	0.00103	0.49(0.625)	0.00007	0.00013	0.57
Vocabulary	−0.00695	0.00253	−2.75^*^	-0.00718	0.00261	−2.75^*^	−0.02007	0.01110	−1.78(0.071)	−0.00284	0.00144	−1.98

*Random intercepts for subjects and items, as well as random slopes for semantic × syntactic interference manipulations were included*.

*A coefficient is a significant predictor of RT or accuracy of comprehension question at p < 0.05 with criterion that |t| or |z| > = 2. The significant predictors are marked with a asterisk and also highlighted in red color*.

These results were from the mixed-effect model analyses including all individual differences predictors. The results of interactions between individual differences measures and semantic/syntactic interference effect are reported in a separate table (see Table 7.)

**Table 7:** **Interaction of semantic/syntactic interference effect and individual differences measures in linear mixed-effects models with all predictors on the reading time and accuracy data**.

**DV**	**Predictor**	**Semantic interference**			**Syntactic interference**		
		**Coefficient**	**SE**	***t*****-score**	**Coefficient**	**SE**	***t*****-score**
Critical (RT)	Category	−0.03348	0.02945	−1.14	0.03008	0.02884	1.04
	WM composite	0.00180	0.00138	1.31	−0.00280	0.00134	−2.09^*^
	Digit	−0.00826	0.01571	−0.53	0.01397	0.01532	0.91
	Stroop	−0.00003	0.00004	−0.72	−0.00002	0.00004	−0.57
	Vocabulary	−0.00002	0.00040	−0.05	0.00037	0.00039	0.93
Spillover (RT)	Category	−0.01438	0.02981	−0.480	−0.00747	0.02780	−0.27
	WM composite	0.00138	0.00140	0.990	−0.00319	0.00129	−2.47^*^
	Digit	−0.00897	0.01596	−0.560	0.03344	0.01474	2.27^*^
	Stroop	0.00004	0.00004	1.050	−0.00001	0.00003	−0.43
	Vocabulary	−0.00015	0.00041	−0.370	−0.00011	0.00038	−0.30
Question (RT)	Category	−0.05930	0.02570	−2.31^*^	−0.00572	0.02348	−0.24
	WM composite	0.00005	0.00120	0.04	0.00030	0.00108	0.28
	Digit	−0.02306	0.01377	−1.67	−0.02018	0.01236	−1.63
	Stroop	−0.00003	0.00003	−0.84	0.00002	0.00003	0.64
	Vocabulary	−0.00036	0.00035	−1.04	0.00037	0.00032	1.15
		**Coefficient**	**SE**	***z*****-score (*****p*****-value)**	**Coefficient**	**SE**	***z*****-score (*****p*****-value)**
Question (Error rates)	Category	−0.03065	0.50950	−0.06(0.95)	0.42810	0.53350	0.80(0.42)
	WM composite	−0.04828	0.02271	−2.13^*^(0.03)	−0.00786	0.02350	−0.34(0.74)
	Digit	0.29550	0.28140	1.05(0.29)	0.25620	0.29130	0.88(0.38)
	Stroop	−0.00057	0.00063	−0.90(0.37)	−0.00002	0.00066	−0.03(0.98)
	Vocabulary	0.01421	0.00669	2.12^*^ (0.03)	0.00632	0.00714	0.89(0.37)

*Vocabulary was included as a control variable. A coefficient is a significant predictor of RT or accuracy of comprehension question at p < 0.05 with criterion that |t| > = 2 or |z| > = 2. The significant predictors are marked with a asterisk and also highlighted in red color*.

**Figure 1:**
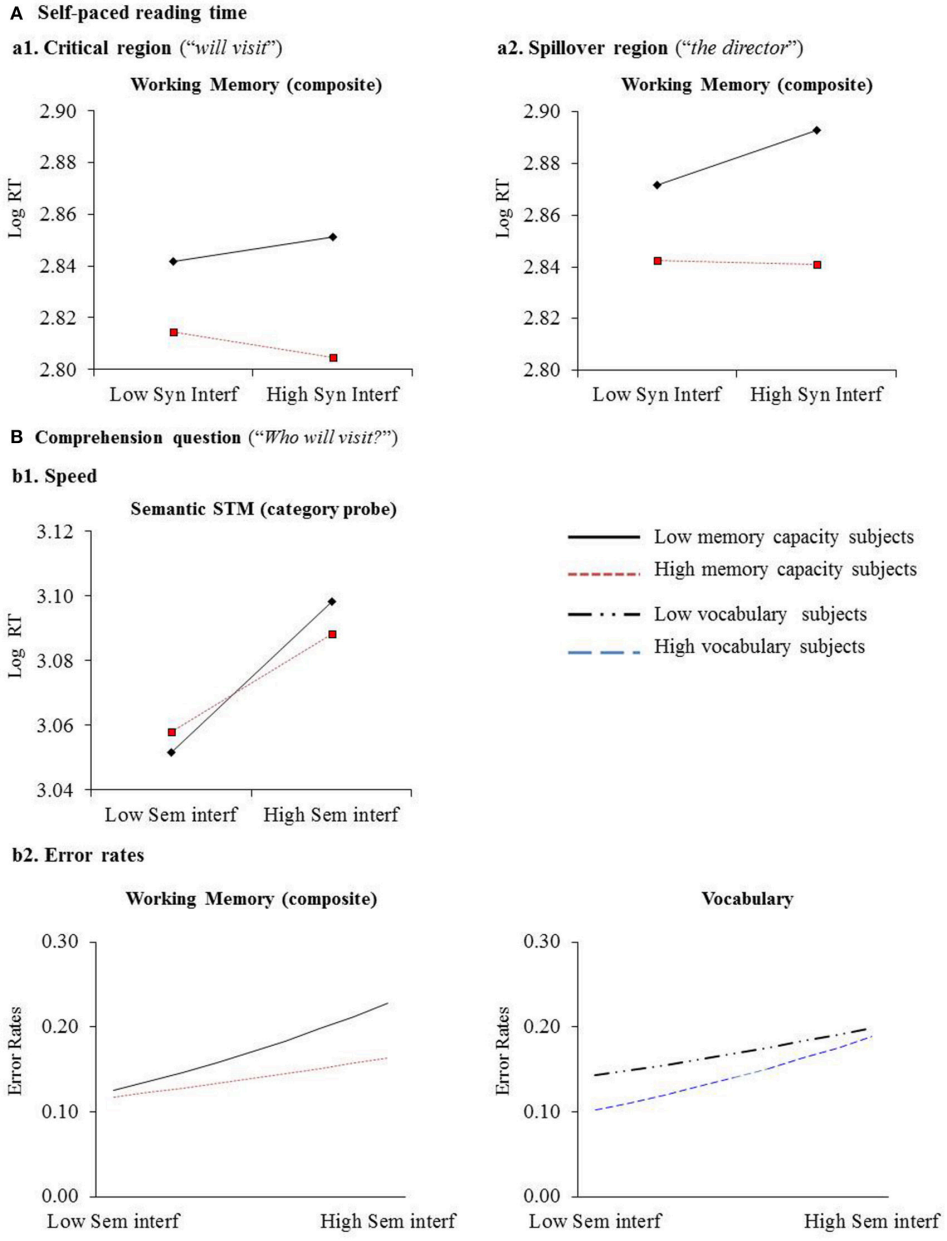
**Significant interactions in mixed-effects analysis for individual differences measure with interference effects (see Dawson, 2014, for plotting methods)**. For RTs **(a1,a2,b1)**, the points plotted for low and high capacity subjects are for −1 and +1 standard deviation from the mean on the composite WM measure or semantic STM measure. For error rates **(b2)**, the values of the interference effect size (on x-axis), ranging from (−1.5) to (1.5) standard deviations from the mean, were calculated in 0.25 std units, with a line fitted to these effects. Panels **(a1,a2)** show the Syntactic interference × WM composite score interaction in self-paced reading time (ms) in the critical region (“*will visit*”) and the spillover region (“*the director*”), respectively. Panel **(b1)** shows the Semantic interference × Category probe interaction in question answering speed. Panel **(b2)** shows the Semantic interference × WM composite and Semantic interference × Vocabulary interactions in question answering error rates. The scatter plots with data points from each subject are shown in Appendix G in Supplementary Material.

With respect to individual differences effects, while the main effect of syntactic interference was not significant in the critical region, there was a significant interaction between syntactic interference and the composite WM measure in this region (*t* = −2.09), which also appeared in the spillover region (*t* = −2.47; See Figure 1 for a plotting of interaction effects using methods outlined by Dawson, 2014). These interactions indicate online effects of general WM capacity, where those with lower spans tended to have more difficulty with syntactic interference than those with higher spans, with the difference between the high and low span subjects being greatest in the high syntactic interference condition. On the other hand, the interactions between semantic interference and the individual differences measures failed to reach significance in either region. There was also a significant interaction between syntactic interference and digit span in the spillover region, which went in the direction opposite that predicted, indicating that subjects with higher digit span showed larger syntactic interference effects (*t* = 2.27). However, we suggest that digit span acted like a suppressor variable in the WM-syntactic interference relation, rather than playing a crucial independent role. This conclusion is based on the grounds that when digit span was included as a single predictor in the mixed-effects model analysis (with vocabulary as a control variable; as shown in Appendix B in Supplementary Material), the interaction between digit span and syntactic interference was not significant in either the critical region (*t* = 0.47) or spillover region (*t* = 1.62^3^), while the interaction between the WM composite and the syntactic interference effect was marginally significant in both the critical (*t* = −1.66) and the spillover region (*t* = −1.99). Therefore, because digit span did not play an independent role and the weight for the composite WM measure became higher when including digit span (i.e., when removing the contribution of phonological retention to the composite WM measure), the influence of digit span fits the definition of a suppressor variable. Last, due to the potential concern that the type of WM task affects the observed relations between WM capacity and other cognitive functions (e.g., Shipstead et al., 2014), we also constructed two other models in which only the reading span or operation span measure was included along with all of the other individual differences measures to investigate whether the specific processing component in each complex span measure (i.e., sentence reading in the reading span task and arithmetic computation in the operation span task) modulated the WM-language relation as reported in some previous studies (Unsworth et al., 2009). The results of these two models were very similar to those from Model 1 in terms of the pattern of main effects and interactions (see Appendices D,E in Supplementary Material for full model output). Although, the relations between reading span and syntactic interference were somewhat stronger than those between operation span and syntactic interference, adding reading span to the model with operation span did not significantly improve the fit (see Appendix F in Supplementary Material). Thus, it appears that the interactions between WM and syntactic interference are due to the overlapping variance between reading span and operation span.

##### Summary of self-paced reading effects

The findings were in line with previous studies using a similar paradigm in showing semantic and syntactic interference effects in self-paced reading measures (Van Dyke and Lewis, 2003; Van Dyke, 2007). More critically, the present study provides the first evidence that the size of the syntactic interference effect is related to a measure of general WM capacity, and this relation was observed at both the critical and the spillover regions. However, despite the fact that a marginal main effect of semantic interference was obtained in the critical region and a significant effect in the spillover region, no interaction between semantic interference and any of the individual differences measures was observed. It should be noted that the significant interactions between WM capacity and syntactic interference could not be attributed to either verbal knowledge (as measured by WAIS vocabulary) or general executive control ability (as measured by Stroop) as these WM effects were significant even though vocabulary and Stroop were included in the models. Neither vocabulary nor Stroop showed significant interactions with syntactic interference. One might question whether the absence of interactions with vocabulary or Stroop resulted from the significant correlations of these measures with the two WM measures, and thus inclusion of the WM measures masked their influence. However, as shown in Appendix B in Supplementary Material (which presents single variable models with vocabulary as a control variable), when vocabulary and Stroop were included in a model without the other STM or WM measures, no significant interactions were observed with syntactic or semantic interference for either measure (all *t*s < 1).

#### Comprehension questions

##### Model 2 (offline measures)

The results of main effects and interactions of both experimental manipulations and individual differences measures are shown in Tables 6, 7. For comprehension question error rates, the mixed-effects analysis revealed significant main effects of both syntactic and semantic interference, with a higher error rate for the HiSyn compared to the LoSyn conditions (0.15 vs. 0.10), and a higher error rate for the HiSem compared to the LoSem conditions (0.15 vs. 0.10). The interaction of semantic and syntactic interference was not significant. For question answering RTs, there was a significant main effect of syntactic interference with slower responses in the HiSyn than the LoSyn condition (1,359 vs. 1,325 ms), and a main effect of semantic interference, with slower responses in the HiSem condition than the LoSem condition (1,411 vs. 1,273 ms). The interaction was not significant.

With respect to the individual differences interactions of primary interest, for RTs, there was a reliable interaction between the semantic interference effect and category probe span (*t* = −2.31)^4^, indicating that participants with better semantic STM were less distracted by a semantically plausible intervening NP in the HiSem conditions (as shown in Figure 1b1), even after controlling for vocabulary. None of the interactions with other individual differences measures was significant. For error rates, the interaction between the composite WM measure and semantic interference was significant (*t* = −2.13, *p* = 0.03), such that those with higher WM capacity showed smaller semantic interference effects. There was also a significant interaction between vocabulary and semantic interference (*t* = 2.12, *p* = 0.03), with those with higher vocabulary scores showing larger interference effects, which was opposite the effect reported in Van Dyke et al. (2014). Both interactions are displayed in Figure 1b2. Unlike the case for digit span, the negative weight for the interaction with vocabulary could not be attributed to a suppressor effect, as the same pattern was obtained in the model with vocabulary as a single predictor (see Appendix B in Supplementary Material). One might postulate that those with larger vocabularies have tighter links among concepts, resulting in greater spreading activation and more interference. It is not transparent why those with greater vocabularies showed larger interference effects in the present study whereas in the Van Dyke et al. (2014) study, those with larger vocabularies were less affected by an interfering external load. Of course, there are many differences in the two studies including the type of manipulation (i.e., external load vs. sentence-internal interference), the sentence structures, and the measure of vocabulary (i.e., expressive vs. receptive vocabulary). Thus, future work would be needed to tease apart the source of the difference pattern of effects for vocabulary.

##### Summary of question answering effects

Robust effects of semantic and syntactic interference were observed for both RTs and error rates in question answering. For question answering speed, an interaction between the semantic STM measure (category probe) and semantic interference was obtained. For question answering error rates, the interaction between semantic interference and the composite WM measure, and the interaction between semantic interference and vocabulary were significant^5^.

## Discussion

Overall, these results provided further evidence demonstrating both syntactic and semantic interference effects during sentence processing (Van Dyke and Lewis, 2003; Van Dyke and McElree, 2006, 2011; Van Dyke, 2007; Glaser et al., 2013; Van Dyke et al., 2014). Participants were slower to read phrases and less accurate and slower to answer comprehension questions with high semantic or syntactic interference.

Of particular interest for the current study are the implications of the interactions between individual differences measures and sensitivity to interference for theories of the relation between WM and sentence processing (see Table 1). Table 8 indicates the significant interactions that were obtained. The discovery of an interaction of syntactic interference with the WM composite in reading times is an important finding, as no study has yet examined individual differences with respect to this type of interference. This finding is inconsistent with both the general WM and language-specific WM approaches. That is, the general WM approach predicted interactions between the composite WM measure and *both* syntactic and semantic interference during online processing and question answering, whereas the language-specific approach would not have predicted WM to be related to either type of interference in online measures.

**Table 8:** **Significant interactions between self-paced reading and individual differences measures**.

	**Self-paced reading**		**Question answering**	
	**RT (critical)**	**RT (spillover)**	**RT (question)**	**Accuracy**
**SYNTACTIC INTERFERENCE**				
× WM Composite	^*^	^*^		
× Category probe				
× Digit span		^*^ (−)		
× Vocabulary				
× Stroop				
**SEMANTIC INTERFERENCE**				
× WM Composite				^*^
× Category probe			^*^	
× Digit span				
× Vocabulary				^*^ (−)
× Stroop				

*Asterisks followed by a minus sign indicate interactions that went in the direction opposite that predicted*.

With respect to the multiple capacities approach, the results were mixed. Consistent with this approach, the category probe measure of semantic STM interacted significantly with semantic but not syntactic interference in question answering RTs, even when a measure of verbal knowledge (i.e., WAIS vocabulary) was included as a control variable. Thus, semantic capacity *per se* beyond semantic knowledge related to semantic interference. Moreover, the measure of phonological retention (digit span) did not interact in the predicted direction with any measure. The specific relation of the composite WM measure to syntactic but not semantic interference sensitivity in self-paced reading might also be seen as consistent with the multiple capacities view, to the extent that the complex span measures tap a retention capacity that is more relevant to syntactic than semantic processing. However, the interaction of the complex span measure, but not the semantic STM measure, with semantic interference in accuracy is inconsistent with this view.

The results with respect to the cue-based parsing approach are also mixed. The significant interaction between syntactic interference and the WM composite in the critical and spillover regions, together with the interaction of complex span with semantic interference in question answering, appear to contradict the assertion that WM capacity *per se* is not involved in sentence processing (e.g., McElree et al., 2003; Van Dyke et al., 2014). The Van Dyke et al. study, which is the only other study to examine individual differences in sensitivity to interference, albeit semantic interference from distractors outside the sentence, did not find an interaction with IQ-partialled complex span tasks, however they did observe an interaction with IQ, which shares significant variance with complex span measures. Thus, it is possible that the effects observed here are tapping the variance shared between WM and IQ. Further research is needed to determine the nature of this variance.

The other primary result in the Van Dyke et al. (2014) study was to emphasize the quality of linguistic representations, as measured by an assessment of receptive vocabulary, as a factor in determining retrieval success. On the basis of this, we hypothesized that verbal knowledge (as assessed by WAIS vocabulary) may be related to either syntactic or semantic interference or both. We found some evidence for such a claim, as we found that vocabulary did interact with semantic interference, though only in questioning answering and with an effect in the opposite direction to that expected (i.e., higher vocabulary subjects showed greater interference). We nevertheless interpret the significant result as supporting the suggestion that qualitative aspects of the to-be-retrieved representations contribute to the size of interference effects. Additionally, a role for another general ability measure related to interference resolution ability (i.e., the Stroop effect) might have been expected on the cue-based parsing approach, but this failed to interact with interference in any dependent measure. This is discussed further below.

An issue for all models is that, as shown in Table 8, the interactions between syntactic interference and capacity measures only appeared during sentence reading, whereas those for semantic interference only appeared during question answering. The failure to find an interaction between semantic interference and any individual differences measure during online processing is somewhat surprising given that a significant 47 ms semantic interference effect was obtained in the spillover region; however, this null interaction with individual differences measures was also reported in an earlier study (Van Dyke et al., 2014). The significant interaction in question answering RT between category probe and semantic interference might be taken to imply that semantic STM or WM capacity only comes into play in *offline semantic* processing (e.g., in reviewing the sentence interpretation before answering a question). However, it is possible that the question answering effects reflect online processes that were begun earlier, but were not complete until past the end of the sentence. This is plausible given that the integration of semantic information appears to be slower than that for syntactic information (McElree and Griffith, 1995, 1998; Boland and Blodgett, 2001; Friederici, 2002; Hagoort, 2003). The difference in time course could be due to the finite set of grammatical features to be considered vs. the more complex considerations involved in determining semantic fit (e.g., “the play arrives” may be plausible even though “play” is inanimate). In addition, an important point to note for the present paradigm is that the correct resolution of semantic interference depends on using discriminative syntactic cues. That is, further consideration of semantic features will not resolve the semantic interference between two nouns if both are equally plausible as the subject of the verb. Therefore, perhaps the later time course for semantic interaction effects occurs because semantic conflict attracts attention to semantic features, whereas resolution of the conflict involves a shift of attention to syntactic features^6^.

### The complexity of WM capacity and implications for sentence processing theories

As discussed in the introduction, early studies relating complex span measures like reading span to sentence processing assumed that these measures reflected a single capacity that could be flexibly allocated to storage or processing (Daneman and Carpenter, 1980; Just and Carpenter, 1992). However, recent studies argue against the assumption that WM capacity reflects a unitary capacity. Both Shipstead et al. (2014) and Unsworth et al. (2014) concluded, on the basis of large scale individual differences studies, that WM capacity can be divided into three components reflecting primary memory capacity (i.e., the capacity for maintaining information in the focus of attention), attentional control, and the ability to retrieve information from outside the focus of attention (i.e., from secondary memory). Secondary memory retrieval ability was not assessed in the present study and thus we cannot comment on its potential contribution to interactions of WM capacity with sensitivity to syntactic and semantic interference. Although, digit span may not be an ideal measure of primary memory capacity, it is highly correlated with other measures that have been argued to reflect this capacity (e.g., running span; Cowan et al., 2005). Thus, the significant interaction of WM capacity with syntactic interference even with digit span in the model suggests that the influence of WM capacity does not reflect the influence of primary memory capacity. An obvious candidate for explaining the relevant shared variance between WM capacity and interference resolution that would be consistent within the cue-based retrieval framework is attentional control. This hypothesis could also explain the link observed between WM capacity as measured by the complex WM tasks and the efficiency of controlled memory retrieval in previous memory studies (Öztekin and McElree, 2010; Mızrak and Öztekin, 2016). Öztekin and McElree found that low-WM span subjects took longer for the controlled retrieval of episodic information as compared to high-WM span subjects. As they suggested that there should be no differences in subjects' primary memory capacity (or the limit of focus of attention) assuming both groups could only maintain 1-item, such a relation might reflect better attentional control for high WM span subjects.

However, no interactions with our measure most related to attentional control—Stroop interference—were observed in the current study. It is possible, though, that the Stroop effect is not the most appropriate measure of this capacity. Of note is the fact that in the Shipstead et al. (2014) study the Stroop effect had the lowest weight on the attentional control factor out of the three variables used to tap that construct (the other two being anti-saccade and flanker tests). Particularly for sentence parsing, the type of inhibition required for Stroop (inhibition of a prepotent response) may not be the same as the inhibition required to resolve interference from incorrectly retrieved information during sentence processing (see Friedman and Miyake, 2004). This may be more consistent with either a mechanism supporting selection from a range of partially matching competitors (e.g., Thompson-Schill et al., 1997; Novick et al., 2005), or reanalysis, involving rejection of an incorrectly retrieved item or incorrectly constructed dependency (Van Dyke and Lewis, 2003). In neither of these cases is there a prepotent response, and so it is perhaps not surprising that we failed to find this association with the Stroop effect. Clearly, however, future work would be needed to show that other measures of interference resolution ability, particularly those involved in the resolution of proactive interference (Friedman and Miyake, 2004; Pettigrew and Martin, 2014), do relate to syntactic and semantic interference effects. Ideally, such a study would also include measures of primary memory capacity and the ability to retrieve information from secondary memory, as cue-based retrieval from outside the focus of attention is a crucial component of the cue-based parsing approach. Thus, a finding that general secondary memory retrieval abilities are related to the resolution of interference in sentence processing would also be supportive of this view.

The relation of category probe span, a STM measure, to semantic interference may suggest that differences in aspects of primary memory do affect interference resolution in a code-specific manner. This result is consistent with finding in the WM literature indicating a role for modality-specific retention abilities, in addition to general WM capacity, in a variety of cognitive domains (See Conway and Kovacs, 2013). As the cue-based parsing approach has argued that primary memory has a capacity fixed to 1–2 items in the current focus of attention (e.g., Lewis et al., 2006), the findings for category probe were not predicted from this theoretical approach. However, within the cue-based parsing framework, this finding could be interpreted as reflecting individual differences in the rate at which semantic features may be reactivated or become lost outside the focus of attention. Moreover, the fact that this measure does not interact with syntactic interference may imply that the rate of feature loss is different for semantic and syntactic information. This view is equivalent to the assumption in the multiple capacities approach of different capacities for semantic and syntactic information—framing it instead in terms of the rate of feature loss instead of buffer capacity (Martin and Romani, 1994; Martin and He, 2004). Thus, we might expect that a separate measure of syntactic retention—if such could be identified—would relate to syntactic interference, with the size of the syntactic interference effect being determined by the rate at which syntactic features are reactivated or lost.

## Conclusions

In line with prior studies, our study demonstrated semantic and syntactic interference from unavailable constituents during sentence processing, consistent with the cue-based parsing approach. The novel aspect of the present study was the investigation of the role of individual differences in WM in modulating these interference effects. We found that general WM capacity derived from complex span tasks showed a relation to syntactic interference during online sentence processing, and to semantic interference during question answering. In addition, a measure of semantic STM capacity predicted the size of semantic but not syntactic interference effects in question answering, while phonological capacity did not predict the size of any interference effects. These interactions with WM were observed in both online and offline processing, even when controlling for vocabulary differences.

The pattern of results argues against claims that a specialized WM is involved in sentence parsing that is different from the capacities tapped by standard simple or complex span measures (Caplan and Waters, 1999). We speculate that the relations to general WM capacity reflect the role controlled attention and potentially secondary memory retrieval involved in both complex span measures and in resolving interference during sentence comprehension. In addition, we consider the specific relation between semantic STM and semantic interference as an indication that code-specific retention capacities mediate resolution and that the rate of loss of semantic or syntactic features (if such could be measured) may differ separately across individuals. This latter assumption is consistent with the multiple capacities approach to WM in which there are separable syntactic and semantic capacities, with the rate of loss of featural information replacing the notion of capacity limits.

## Limitations and future directions

There are limitations to the current study, which could be addressed by future research. Most of these were mentioned earlier, but will be summarized here. First, the multiple capacities approach assumes a separable syntactic capacity, but no specific measure of that capacity was included here. In future work, it would be important to include a measure of the ability to retain syntactic information *per se*, which would have to be demonstrated to be separable from semantic retention and general working memory capacity. Second, with respect to the cue-based parsing approach, despite the proposal that WM capacity may be so limited as to be irrelevant for parsing, we did nevertheless observe significant relationships between our measure of general WM capacity and comprehension. In the face of data pointing to a highly limited WM capacity for sentence processing (e.g., McElree et al., 2003; Johns et al., 2015), future research will need to address the question of what these measures represent, if not capacity. We have noted that recent research with WM capacity as measured by complex span tasks has suggested that separate mechanisms of maintenance in primary memory (which seems equivalent to the focus of attention), attentional control, and retrieval from long-term memory (e.g., Shipstead et al., 2014; Unsworth et al., 2014) underlie this construct. Future work will need to sort out which of these is the source of the relation between general WM and sentence processing observed here. Here we suggested that attentional control may underlie our findings, but did not measure this capacity directly. This would be important to do. In particular, it will be important to test whether effects of WM capacity would disappear when a measure of the ability to resolve proactive interference (which is distinct from the ability to resolve response/distractor interference as in Stroop; Friedman and Miyake, 2004) is modeled. Finally, given the differences between some of the results obtained here and those reported elsewhere (such as the influence of vocabulary on the direction of interference effects), future work will be needed to understand the extent to which the relationship between WM, vocabulary, and interference resolution depends on the type of task (dual-task vs. standard reading) or the location of distractors (sentence-internal vs. sentence-external).

## Ethics statement

This study was carried out in accordance with the recommendations of the Rice University Institutional Review Board with written informed consent from all subjects. All subjects gave written informed consent in accordance with the Declaration of Helsinki. The protocol was approved by the Rice University Institutional Review Board.

## Author contributions

Conception or design of the work: YT, RM, and JV. Data collection: YT. Data analysis and interpretation: YT, RM, and JV. Drafting the article: YT and RM. Critical revision of the article: YT, RM, and JV. Final approval of the version to be published: YT, RM, and JV.

### Conflict of interest statement

The authors declare that the research was conducted in the absence of any commercial or financial relationships that could be construed as a potential conflict of interest. The reviewer CH and the handling Editor declared their shared affiliation, and the handling Editor states that the process nevertheless met the standards of a fair and objective review.

## Footnotes

^1^We have also conducted the analysis with length residualized reading times (Trueswell et al., 1994). The residual RT was calculated for each participant by subtracting the RT predicted by region length from the actual reading time. In the mixed-effects model analysis, the results from models with residual RTs as dependent variables were very similar to the results from models with log-transformed RTs as dependent variables and region length as fixed effect. Thus, we only reported the results of including word length as fixed effect and log-transformed RT as dependent variables here.

^2^Given that these analyses were intended to determine if collinearity had hidden the contribution of some of the variables, a liberal criterion for significance was used without correction for multiple comparisons.

^3^According to the Student's t-distribution, when *p* = 0.10, *t*_(120)_ = 1.658, *t*_(∞)_ = 1.645. Although, the current mixed-effects model results could only estimate the degrees of freedom, it should be a value between 120 and ∞.

^4^To make the results more comprehensible in that the interpretation of the direction of coefficients would be the same for RTs and accuracy, we used error rates rather than accuracy in the analyses. Therefore, a negative coefficient for the interaction between error rates and the individual differences measures indicates that participants with better ability were more resistant to interference.

^5^While the main effects of the individual differences measures were not a focus of this study, there were some findings of interest. For online sentence comprehension, there was a main effect of vocabulary, indicating that subjects with better vocabularies read more quickly overall (critical verb region, *t* = −2.75; spillover region, *t* = −2.75), consistent with previous studies showing that high-vocabulary subjects are generally more efficient in sentence reading (Perfetti, 2007; Traxler and Tooley, 2007; Kuperman and Van Dyke, 2011; Van Dyke and Shankweiler, 2013). In contrast, a positive effect of category probe was found (critical verb region, *t* = 3.27; spillover region, *t* = 2.18), indicating that subjects with better semantic STM read slower than subjects with poorer semantic STM for all conditions, possibly because the subjects with better semantic STM were more likely to detect the presence of two preceding nouns that might serve as the matrix verb subject. The main effects of digit span, Stroop, and the composite WM measure were not significant. For offline measures, only a significant main effect of category probe was observed in the accuracy data, indicating that individuals with better semantic STM capacity were more accurate in answering comprehension questions overall (*z* = 2.53, *p* = 0.01).

^6^The resolution of semantic interference depends on using discriminative syntactic cues. For example, in the high-semantic, low-syntactic interference condition, the syntactic feature of subject or object is sufficient to determine the correct noun. While in the high-semantic, high-syntactic interference condition where both nouns are subjects, the appropriate noun can only be selected based on its not having been assigned as the subject of another verb. In contrast, for syntactic interference, syntactic conflict may mainly attract attention to syntactic features and it is these features that are needed to resolve the conflict. Therefore, the attention shift from semantic features to syntactic features results in delayed semantic interference resolution as compared to syntactic interference resolution, during which no attention shift is necessary. However, it should be noted that in many other sentence types, semantic and syntactic processes are relatively independent and separable. The processing of semantic information does not always depend on the processing of syntactic information (e.g., Kim and Osterhout, 2005).
