# Supplementary material for: Semantic and Syntactic Interference in Sentence Comprehension: A Comparison of Working Memory Models
Source: Front Psychol. 2017 Feb 15;8:198. doi: 10.3389/fpsyg.2017.00198 (PMC5309252; doi:10.3389/fpsyg.2017.00198)

# Appendix A

Experimental Sentences

| Item | cond | Sentence | Question |
| --- | --- | --- | --- |
| 1 | 1 | The client who had arrived after the important meeting that day was waiting in the office. | Who was waiting? |
| 1 | 2 | The client who had arrived after the important visitor that day was waiting in the office. | Who was waiting? |
| 1 | 3 | The client who implied that the meeting was important that day was waiting in the office. | Who was waiting? |
| 1 | 4 | The client who implied that the visitor was important that day was waiting in the office. | Who was waiting? |
| 2 | 1 | The resident who was living near the dangerous warehouse last month had complained about the investigation. | Who had complained? |
| 2 | 2 | The resident who was living near the dangerous neighbor last month had complained about the investigation. | Who had complained? |
| 2 | 3 | The resident who said that the warehouse was dangerous last month had complained about the investigation. | Who had complained? |
| 2 | 4 | The resident who said that the neighbor was dangerous last month had complained about the investigation. | Who had complained? |
| 3 | 1 | The teacher who was designing the new curriculum last night will come to the office. | Who will come? |
| 3 | 2 | The teacher who was meeting with the new specialist last night will come to the office. | Who will come? |
| 3 | 3 | The teacher who realized that the curriculum was new last night will come to the office. | Who will come? |
| 3 | 4 | The teacher who realized that the specialist was new last night will come to the office. | Who will come? |
| 4 | 1 | The ambassador who had exposed the known conspiracy during the meeting will arrive this morning. | Who will arrive? |
| 4 | 2 | The ambassador who had criticized the known terrorist during the meeting will arrive this morning. | Who will arrive? |
| 4 | 3 | The ambassador who claimed that the conspiracy was known in the meeting will arrive this morning. | Who will arrive? |
| 4 | 4 | The ambassador who claimed that the terrorist was known in the meeting will arrive this morning. | Who will arrive? |
| 5 | 1 | The critic who had enjoyed the memorable play at the new theater will praise the director. | Who will visit? |
| 5 | 2 | The critic who had enjoyed the memorable actress at the new theater will praise the director. | Who will visit? |
| 5 | 3 | The critic who mentioned that the play was memorable at the new theater will praise the director. | Who will visit? |
| 5 | 4 | The critic who mentioned that the actress was memorable at the new theater will praise the director. | Who will visit? |
| 6 | 1 | The unit which was searching for the captured supplies after the bombing will contact the base. | Who will contact the base? |
| 6 | 2 | The unit which was searching for the captured pilots after the bombing will contact the base. | Who will contact the base? |
| 6 | 3 | The unit which suspected that the supplies were captured after the bombing will contact the base. | Who will contact the base? |
| 6 | 4 | The unit which suspected that the pilots were captured after the bombing will contact the base. | Who will contact the base? |
| 7 | 1 | The opponent who was fighting the corrupt government for nearly three year should be arrested immediately. | Who should be arrested? |
| 7 | 2 | The opponent who was fighting the corrupt governor for nearly three year should be arrested immediately. | Who should be arrested? |
| 7 | 3 | The opponent who had claimed that the government was corrupt for nearly three year should be arrested immediately. | Who should be arrested? |
| 7 | 4 | The opponent who had claimed that the governor was corrupt for nearly three year should be arrested immediately. | Who should be arrested? |
| 8 | 1 | The policeman who had found the missing money accidentally was expecting an investigation. | Who was expecting an investigation? |
| 8 | 2 | The policeman who had found the missing boy accidentally was expecting an investigation. | Who was expecting an investigation? |
| 8 | 3 | The policeman who had discovered that the money was missing accidentally was expecting an investigation. | Who was expecting an investigation? |
| 8 | 4 | The policeman who had discovered that the boy was missing accidentally was expecting an investigation. | Who was expecting an investigation? |
| 9 | 1 | The couple who had looked for the cheaper house since the wedding was making a mistake. | Who was making a mistake? |
| 9 | 2 | The couple who had looked for the cheaper agent since the wedding was making a mistake. | Who was making a mistake? |
| 9 | 3 | The couple who thought that the house was cheaper after the wedding was making a mistake. | Who was making a mistake? |
| 9 | 4 | The couple who thought that the agent was cheaper after the wedding was making a mistake. | Who was making a mistake? |
| 10 | 1 | The manager who liked the clever show at the opening ceremony could negotiate a good deal. | Who could negotiate? |
| 10 | 2 | The manager who liked the clever producer at the opening ceremony could negotiate a good deal. | Who could negotiate? |
| 10 | 3 | The manager who said that the show was clever at the opening ceremony could negotiate a good deal. | Who could negotiate? |
| 10 | 4 | The manager who said that the producer was clever at the opening ceremony could negotiate a good deal. | Who could negotiate? |
| 11 | 1 | The suspect who was aware of the unguarded money outside was sleeping during the crime. | Who was sleeping? |
| 11 | 2 | The suspect who was aware of the unguarded teller outside was sleeping during the crime. | Who was sleeping? |
| 11 | 3 | The suspect who knew that the money was unguarded outside was sleeping during the crime. | Who was sleeping? |
| 11 | 4 | The suspect who knew that the teller was unguarded outside was sleeping during the crime. | Who was sleeping? |
| 12 | 1 | The procedures which were favored by the designated laws in California were enacted throughout the country. | what were enacted? |
| 12 | 2 | The procedures which were favored by the designated voters in California were enacted throughout the country. | what were enacted? |
| 12 | 3 | The procedures which ensure that the laws are designated in California were enacted throughout the country. | what were enacted? |
| 12 | 4 | The procedures which ensure that the voters are designated in California were enacted throughout the country. | what were enacted? |
| 13 | 1 | The company which had created the controversial product last year asked for feedback. | Who asks for feedback? |
| 13 | 2 | The company which had hired the controversial spokesman last year asked for feedback. | Who asks for feedback? |
| 13 | 3 | The company that admited that the product is controversial last year asked for feedback. | Who asks for feedback? |
| 13 | 4 | The company that admited that the spokesman is controversial last year asked for feedback. | Who asks for feedback? |
| 14 | 1 | The student who was tried of the demanding assignment at school was leaving for home. | Who was leaving? |
| 14 | 2 | The student who was tried of the demanding coach at school was leaving for home. | Who was leaving? |
| 14 | 3 | The student who said that the assignment was demanding at school was leaving for home. | Who was leaving? |
| 14 | 4 | The student who said that the coach was demanding at school was leaving for home. | Who was leaving? |
| 15 | 1 | The handyman who worked on the ambitious project for the company was standing outside. | Who was standing outside? |
| 15 | 2 | The handyman who worked with the ambitious foreman for the company was standing outside. | Who was standing outside? |
| 15 | 3 | The handyman who said that the project was ambitious to the company was standing outside. | Who was standing outside? |
| 15 | 4 | The handyman who said that the foreman was ambitious to the company was standing outside. | Who was standing outside? |
| 16 | 1 | The witness who will support the fraudulent case at the court will testify against the suspect. | Who will testify? |
| 16 | 2 | The witness who will support the fraudulent defendant at the court will testify against the suspect. | Who will testify? |
| 16 | 3 | The witness who suggested that the case was fraudulent at the court will testify against the suspect. | Who will testify? |
| 16 | 4 | The witness who suggested that the defendant was fraudulent at the court will testify against the suspect. | Who will testify? |
| 17 | 1 | The professor who agreed on the inappropriate reprimand yesterday was acting rudely. | Who was acting rudely? |
| 17 | 2 | The professor who agreed with the inappropriate chairman yesterday was acting rudely. | Who was acting rudely? |
| 17 | 3 | The professor who agreed that the reprimand was inappropriate yesterday was acting rudely. | Who was acting rudely? |
| 17 | 4 | The professor who agreed that the chairman was inappropriate yesterday was acting rudely. | Who was acting rudely? |
| 18 | 1 | The couple who had requested the sophisticated vase from the store was talking about the price. | Who was talking? |
| 18 | 2 | The couple who had requested a sophisticated salesman from the store was talking about the price. | Who was talking? |
| 18 | 3 | The couple who remarked that the vase was sophisticated at the store was talking about the price. | Who was talking? |
| 18 | 4 | The couple who remarked that the salesman was sophisticated at the store was talking about the price. | Who was talking? |
| 19 | 1 | The passenger who was sitting in the new seat on the bus was talking on the phone. | Who was talking? |
| 19 | 2 | The passenger who was sitting behind the new driver on the bus was talking on the phone. | Who was talking? |
| 19 | 3 | The passenger who commented that the seat was new on the bus was talking on the phone. | Who was talking? |
| 19 | 4 | The passenger who commented that the driver was new on the bus was talking on the phone. | Who was talking? |
| 20 | 1 | The physicist who had admired the amazing calculation at the conference was making too much noise. | Who was making noise? |
| 20 | 2 | The physicist who had admired the amazing chemist at the conference was making too much noise. | Who was making noise? |
| 20 | 3 | The physicist who shouted that the calculation was amazing at the conference was making too much noise. | Who was making noise? |
| 20 | 4 | The physicist who shouted that the chemist was amazing at the conference was making too much noise. | Who was making noise? |
| 21 | 1 | The attorney who was questioning the unusual motion in the courtroom was exaggerating quite a bit. | Who was exaggerating? |
| 21 | 2 | The attorney who was questioning the unusual witness in the courtroom was exaggerating quite a bit. | Who was exaggerating? |
| 21 | 3 | The attorney who commented that the motion was unusual in the courtroom was exaggerating quite a bit. | Who was exaggerating? |
| 21 | 4 | The attorney who commented that the witness was unusual in the courtroom was exaggerating quite a bit. | Who was exaggerating? |
| 22 | 1 | The candidate who was attacked by the dishonest commercial in the newspaper was losing the race. | Who was losing? |
| 22 | 2 | The candidate who was attacked by the dishonest senator in the newspaper was losing the race. | Who was losing? |
| 22 | 3 | The candidate who charged that the commercial was dishonest in the newspaper was losing the race. | Who was losing? |
| 22 | 4 | The candidate who charged that the senator was dishonest in the newspaper was losing the race. | Who was losing? |
| 23 | 1 | The publicist who had paid for the brilliant painting at the first meeting will cancel the exhibit. | Who will cancel the exhibit? |
| 23 | 2 | The publicist who had paid for the brilliant painter at the first meeting will cancel the exhibit. | Who will cancel the exhibit? |
| 23 | 3 | The publicist who assumed that the painting was brilliant at the first meeting will cancel the exhibit. | Who will cancel the exhibit? |
| 23 | 4 | The publicist who assumed that the painter was brilliant at the first meeting will cancel the exhibit. | Who will cancel the exhibit? |
| 24 | 1 | The judge who had criticized the questionable evidence recently had misunderstood the facts. | Who had misunderstood? |
| 24 | 2 | The judge who had criticized the questionable witness recently had misunderstood the facts. | Who had misunderstood? |
| 24 | 3 | The judge who decided that the evidence was questionable recently had misunderstood the facts. | Who had misunderstood? |
| 24 | 4 | The judge who decided that the witness was questionable recently had misunderstood the facts. | Who had misunderstood? |
| 25 | 1 | The secretary who was complaining about the unreasonable policy on TV is quitting next month. | Who is quitting? |
| 25 | 2 | The secretary who was complaining about the unreasonable director on TV is quitting next month. | Who is quitting? |
| 25 | 3 | The secretary who complains that the policy is unreasonable on TV is quitting next month. | Who is quitting? |
| 25 | 4 | The secretary who complains that the director is unreasonable on TV is quitting next month. | Who is quitting? |
| 26 | 1 | The girl who was complaining about the annoying pain to everybody had visited the clinic. | Who had visited the clinic? |
| 26 | 2 | The girl who was complaining about the annoying mother to everybody had visited the clinic. | Who had visited the clinic? |
| 26 | 3 | The girl who said that the pain was annoying to everybody had visited the clinic. | Who had visited the clinic? |
| 26 | 4 | The girl who said that the mother was annoying to everybody had visited the clinic. | Who had visited the clinic? |
| 27 | 1 | The director who disliked the outrageous performance in the movie had wanted to quit. | Who wanted to quit? |
| 27 | 2 | The director who disliked the outrageous performer in the movie had wanted to quit. | Who wanted to quit? |
| 27 | 3 | The director who exclaimed that the performance was outrageous in the movie had wanted to quit. | Who wanted to quit? |
| 27 | 4 | The director who exclaimed that the performer was outrageous in the movie had wanted to quit. | Who wanted to quit? |
| 28 | 1 | The informant who had been exposing the illegal company to the public was arrested last night. | Who was arrested? |
| 28 | 2 | The informant who had been exposing the illegal immigrant to the public was arrested last night. | Who was arrested? |
| 28 | 3 | The informant who explained that the company was illegal to the public was arrested last night. | Who was arrested? |
| 28 | 4 | The informant who explained that the immigrant was illegal to the public was arrested last night. | Who was arrested? |
| 29 | 1 | The experts who were apologizing for the biased story during class will explain the decision. | Who will explain? |
| 29 | 2 | The experts who were apologizing for the biased judge during class will explain the decision. | Who will explain? |
| 29 | 3 | The experts who admitted that the story was biased during class will explain the decision. | Who will explain? |
| 29 | 4 | The experts who admitted that the judge was biased during class will explain the decision. | Who will explain? |
| 30 | 1 | The thief who had stolen from the strict church for a while lived near the sanctuary. | Who lived nearby? |
| 30 | 2 | The thief who had stolen from the strict nun for a while lived near the sanctuary. | Who lived nearby? |
| 30 | 3 | The thief who had known that the church was strict for a while lived near the sanctuary. | Who lived nearby? |
| 30 | 4 | The thief who had known that the nun was strict for a while lived near the sanctuary. | Who lived nearby? |
| 31 | 1 | The child who was playing with the dangerous toy all day long was running toward the park. | Who was running? |
| 31 | 2 | The child who was playing with the dangerous stranger all day long was running toward the park. | Who was running? |
| 31 | 3 | The child who wondered if the toy was dangerous all day long was running toward the park. | Who was running? |
| 31 | 4 | The child who wondered if the stranger was dangerous all day long was running toward the park. | Who was running? |
| 32 | 1 | The owner who had driven out the new undergrowth last summer had made mistakes. | Who had made mistakes? |
| 32 | 2 | The owner who had driven out the new assistant last summer had made mistakes. | Who had made mistakes? |
| 32 | 3 | The owner who regretted that the undergrowth was new last summer had made mistakes. | Who had made mistakes? |
| 32 | 4 | The owner who regretted that the assistant was new last summer had made mistakes. | Who had made mistakes? |
| 33 | 1 | The student who had merely seen the loud party last time was drinking under age. | Who was drinking? |
| 33 | 2 | The student who had merely seen the loud partygoer last time was drinking under age. | Who was drinking? |
| 33 | 3 | The student who testified that the party was loud last time was drinking under age. | Who was drinking? |
| 33 | 4 | The student who testified that the partygoer was loud last time was drinking under age. | Who was drinking? |
| 34 | 1 | The enemy who had shot at the hidden tank in the garage was lying in the bushes. | Who was lying in the bushes? |
| 34 | 2 | The enemy who had shot at the hidden man in the garage was lying in the bushes. | Who was lying in the bushes? |
| 34 | 3 | The enemy who saw that the tank was hidden in the garage was lying in the bushes. | Who was lying in the bushes? |
| 34 | 4 | The enemy who saw that the man was hidden in the garage was lying in the bushes. | Who was lying in the bushes? |
| 35 | 1 | The burglar who had stolen the precious jewel from the store was scared by the alarm. | Who was scared? |
| 35 | 2 | The burglar who had stolen the precious baby from the store was scared by the alarm. | Who was scared? |
| 35 | 3 | The burglar who thought that the jewel was precious at the store was scared by the alarm. | Who was scared? |
| 35 | 4 | The burglar who thought that the baby was precious at the store was scared by the alarm. | Who was scared? |
| 36 | 1 | The salesman who had upset the alcoholic drink last night will complain to the manager. | Who will complain? |
| 36 | 2 | The salesman who had upset the alcoholic waiter last night will complain to the manager. | Who will complain? |
| 36 | 3 | The salesman who thought that the drink was alcoholic last night will complain to the manager. | Who will complain? |
| 36 | 4 | The salesman who thought that the waiter was alcoholic last night will complain to the manager. | Who will complain? |
| 37 | 1 | The doorman who had denounced the dreadful crime last month was planning something awful. | Who was planning something awful? |
| 37 | 2 | The doorman who had denounced the dreadful criminal last month was planning something awful. | Who was planning something awful? |
| 37 | 3 | The doorman who remarked that the crime was dreadful last month was planning something awful. | Who was planning something awful? |
| 37 | 4 | The doorman who remarked that the criminal was dreadful last month was planning something awful. | Who was planning something awful? |
| 38 | 1 | The hostess who had yelled about the dirty room loudly will forget about the mess. | Who will forget about the mess? |
| 38 | 2 | The hostess who had yelled about the dirty toddler loudly will forget about the mess. | Who will forget about the mess? |
| 38 | 3 | The hostess who yelled that the room was dirty loudly will forget about the mess. | Who will forget about the mess? |
| 38 | 4 | The hostess who yelled that the toddler was dirty loudly will forget about the mess. | Who will forget about the mess? |
| 39 | 1 | The secretary who had answered the angry calls this Monday should apologize to the company. | Who should apologize? |
| 39 | 2 | The secretary who had answered the angry businessmen this Monday should apologize to the company. | Who should apologize? |
| 39 | 3 | The secretary who claimed that the calls were angry this Monday should apologize to the company. | Who should apologize? |
| 39 | 4 | The secretary who claimed that the businessmen were angry this Monday should apologize to the company. | Who should apologize? |
| 40 | 1 | The teacher who had graded the difficult test last semester could answer the question. | Who could answer the question? |
| 40 | 2 | The teacher who had graded the difficult child last semester could answer the question. | Who could answer the question? |
| 40 | 3 | The teacher who disagreed that the test was difficult last semester could answer the question. | Who could answer the question? |
| 40 | 4 | The teacher who disagreed that the child was difficult last semester could answer the question. | Who could answer the question? |
| 41 | 1 | The teller who was working in the boring room in the back building will quit the job. | Who will quit? |
| 41 | 2 | The teller who was working for the boring boss in the back building will quit the job. | Who will quit? |
| 41 | 3 | The teller who felt that the room was boring in the back building will quit the job. | Who will quit? |
| 41 | 4 | The teller who felt that the boss was boring in the back building will quit the job. | Who will quit? |
| 42 | 1 | The subject who had argued about the difficult quiz on the school message board will leave soon. | Who will leave soon? |
| 42 | 2 | The subject who had argued about the difficult professor on the school message board will leave soon. | Who will leave soon? |
| 42 | 3 | The subject who learned that the quiz was difficult on the school message board will leave soon. | Who will leave soon? |
| 42 | 4 | The subject who learned that the professor was difficult on the school message board will leave soon. | Who will leave soon? |
| 43 | 1 | The waitress who had presented the greasy menu last time was unconcerned about the first impression. | Who was unconcerned? |
| 43 | 2 | The waitress who had presented the greasy cook last time was unconcerned about the first impression. | Who was unconcerned? |
| 43 | 3 | The waitress who knew that the menu was greasy last time was unconcerned about the first impression. | Who was unconcerned? |
| 43 | 4 | The waitress who knew that the cook was greasy last time was unconcerned about the first impression. | Who was unconcerned? |
| 44 | 1 | The boy who had dumped the rich soil last week should apologize for the mistake. | Who should apologize? |
| 44 | 2 | The boy who had dumped the rich girl last week should apologize for the mistake. | Who should apologize? |
| 44 | 3 | The boy who ignored that the soil was rich last week should apologize for the mistake. | Who should apologize? |
| 44 | 4 | The boy who ignored that the girl was rich last week should apologize for the mistake. | Who should apologize? |
| 45 | 1 | The mother who had dropped off the expensive bill recently will appreciate the discount. | Who will appreciate the discount? |
| 45 | 2 | The mother who had dropped off the expensive teenager recently will appreciate the discount. | Who will appreciate the discount? |
| 45 | 3 | The mother who remarked that the bill was expensive recently will appreciate the discount. | Who will appreciate the discount? |
| 45 | 4 | The mother who remarked that the teenager was expensive recently will appreciate the discount. | Who will appreciate the discount? |
| 46 | 1 | The audience who was watching the outrageous show last night will remember the jokes. | Who will remember the jokes? |
| 46 | 2 | The audience who was watching the outrageous comedian last night will remember the jokes. | Who will remember the jokes? |
| 46 | 3 | The audience who thought that the show was outrageous last night will remember the jokes. | Who will remember the jokes? |
| 46 | 4 | The audience who thought that the comedian was outrageous last night will remember the jokes. | Who will remember the jokes? |
| 47 | 1 | The student who appreciated the helpful advise in the counseling session will make the right decision. | Who will make the right decision? |
| 47 | 2 | The student who appreciated the helpful advisor in the counseling session will make the right decision. | Who will make the right decision? |
| 47 | 3 | The student who mentioned that the advice was helpful in the counseling session will make the right decision. | Who will make the right decision? |
| 47 | 4 | The student who mentioned that the advisor was helpful in the counseling session will make the right decision. | Who will make the right decision? |
| 48 | 1 | The actress who had gotten the popular award in Europe was recognized in public. | Who was recognized? |
| 48 | 2 | The actress who had gotten the popular boyfriend in Europe was recognized in public. | Who was recognized? |
| 48 | 3 | The actress who loved that the award was popular in Europe was recognized in public. | Who was recognized? |
| 48 | 4 | The actress who loved that the boyfriend was popular in Europe was recognized in public. | Who was recognized? |
| 49 | 1 | The hostess who had ignored the messy table after the party was talking to some friends. | Who was talking? |
| 49 | 2 | The hostess who had ignored the messy guest after the party was talking to some friends. | Who was talking? |
| 49 | 3 | The hostess who ignored that the table was messy after the party was talking to some friends. | Who was talking? |
| 49 | 4 | The hostess who ignored that the guest was messy after the party was talking to some friends. | Who was talking? |
| 50 | 1 | The teacher who was teaching the Korean alphabet at school will translate the vocabulary. | Who will translate? |
| 50 | 2 | The teacher who was teaching the Korean child at school will translate the vocabulary. | Who will translate? |
| 50 | 3 | The teacher who knew that the alphabet was Korean at school will translate the vocabulary. | Who will translate? |
| 50 | 4 | The teacher who knew that the child was Korean at school will translate the vocabulary. | Who will translate? |
| 51 | 1 | The captain who was watching the beautiful sunset on the boat was steering the ship. | Who was steering? |
| 51 | 2 | The captain who was watching the beautiful woman on the boat was steering the ship. | Who was steering? |
| 51 | 3 | The captain who saw that the sunset was beautiful on the boat was steering the ship. | Who was steering? |
| 51 | 4 | The captain who saw that the woman was beautiful on the boat was steering the ship. | Who was steering? |
| 52 | 1 | The boy who was seeing the silly movie at the theater was wasting money. | Who was wasting money? |
| 52 | 2 | The boy who was seeing the silly girl at the theater was wasting money. | Who was wasting money? |
| 52 | 3 | The boy who disagreed that the movie was silly at the theater was wasting money. | Who was wasting money? |
| 52 | 4 | The boy who disagreed that the girl was silly at the theater was wasting money. | Who was wasting money? |
| 53 | 1 | The customer who had asked about the expensive program yesterday should buy the computer. | Who should buy the computer? |
| 53 | 2 | The customer who had asked about the expensive programmer yesterday should buy the computer. | Who should buy the computer? |
| 53 | 3 | The customer who forgot that the program was expensive yesterday should buy the computer. | Who should buy the computer? |
| 53 | 4 | The customer who forgot that the programmer was expensive yesterday should buy the computer. | Who should buy the computer? |
| 54 | 1 | The prince who had saved the beautiful castle last time had forgotten the magic wand. | Who had forgotten the wand? |
| 54 | 2 | The prince who had saved the beautiful princess last time had forgotten the magic wand. | Who had forgotten the wand? |
| 54 | 3 | The prince who thought that the castle was beautiful last time had forgotten the magic wand. | Who had forgotten the wand? |
| 54 | 4 | The prince who thought that the princess was beautiful last time had forgotten the magic wand. | Who had forgotten the wand? |
| 55 | 1 | The housekeeper who had told about the secret room to the media was fired for the error. | Who was fired? |
| 55 | 2 | The housekeeper who had told about the secret accountant to the media was fired for the error. | Who was fired? |
| 55 | 3 | The housekeeper who revealed that the room was a secret to the media was fired for the error. | Who was fired? |
| 55 | 4 | The housekeeper who revealed that the accountant was a secret to the media was fired for the error. | Who was fired? |
| 56 | 1 | The batter who had swung at the tiny ball at the last game should play baseball. | Who should play? |
| 56 | 2 | The batter who had swung at the tiny pitcher at the last game should play baseball. | Who should play? |
| 56 | 3 | The batter who said that the ball was tiny at the last game should play baseball. | Who should play? |
| 56 | 4 | The batter who said that the pitcher was tiny at the last game should play baseball. | Who should play? |
| 57 | 1 | The traveler who had dreamt about the dark shadow last night will win the lottery. | Who will win? |
| 57 | 2 | The traveler who had dreamt about the dark man last night will win the lottery. | Who will win? |
| 57 | 3 | The traveler who remembered that the shadow was dark last night will win the lottery. | Who will win? |
| 57 | 4 | The traveler who remembered that the man was dark last night will win the lottery. | Who will win? |
| 58 | 1 | The employer who had criticized the old computer at the company meeting will regret the nasty comment. | Who will regret? |
| 58 | 2 | The employer who had criticized the old employee at the company meeting will regret the nasty comment. | Who will regret? |
| 58 | 3 | The employer who remarked that the computer was old at the company meeting will regret the nasty comment. | Who will regret? |
| 58 | 4 | The employer who remarked that the employee was old at the company meeting will regret the nasty comment. | Who will regret? |
| 59 | 1 | The family which had enjoyed the offensive music at the concert was ignorant about quality performances. | Who was ignorant? |
| 59 | 2 | The family which had enjoyed the offensive singer at the concert was ignorant about quality performances. | Who was ignorant? |
| 59 | 3 | The family which had disregarded that the music was offensive at the concert was ignorant about quality performances. | Who was ignorant? |
| 59 | 4 | The family which had disregarded that the singer was offensive at the concert was ignorant about quality performances. | Who was ignorant? |
| 60 | 1 | The passenger who had hidden the dangerous explosive in the back room will leave immediately. | Who will leave immediately? |
| 60 | 2 | The passenger who had hidden the dangerous criminal in the back room will leave immediately. | Who will leave immediately? |
| 60 | 3 | The passenger who understood that the explosive was dangerous in the back room will leave immediately. | Who will leave immediately? |
| 60 | 4 | The passenger who understood that the criminal was dangerous in the back room will leave immediately. | Who will leave immediately? |
| 61 | 1 | The biologist who was testing the contaminated water in the village was afraid of dying. | Who was afraid? |
| 61 | 2 | The biologist who was testing the contaminated animal in the village was afraid of dying. | Who was afraid? |
| 61 | 3 | The biologist who disliked that the water was contaminated in the village was afraid of dying. | Who was afraid? |
| 61 | 4 | The biologist who disliked that the animal was contaminated in the village was afraid of dying. | Who was afraid? |
| 62 | 1 | The mother who had punished the bad dog last week will explain the reason. | Who will explain the reason? |
| 62 | 2 | The mother who had punished the bad boy last week will explain the reason. | Who will explain the reason? |
| 62 | 3 | The mother who thought that the dog was bad last week will explain the reason. | Who will explain the reason? |
| 62 | 4 | The mother who thought that the boy was bad last week will explain the reason. | Who will explain the reason? |
| 63 | 1 | The hero who had saved the frightened cat from the burning car could explain the accident. | Who could explain the accident? |
| 63 | 2 | The hero who had saved the frightened lady from the burning car could explain the accident. | Who could explain the accident? |
| 63 | 3 | The hero who saw that the cat was frightened of the burning car could explain the accident. | Who could explain the accident? |
| 63 | 4 | The hero who saw that the lady was frightened of the burning car could explain the accident. | Who could explain the accident? |
| 64 | 1 | The boy who had stolen the old trophy that night should stop stealing. | Who should stop stealing? |
| 64 | 2 | The boy who had stolen from the old man that night should stop stealing. | Who should stop stealing? |
| 64 | 3 | The boy who knew that the trophy was old that night should stop stealing. | Who should stop stealing? |
| 64 | 4 | The boy who knew that the man was old that night should stop stealing. | Who should stop stealing? |
| 65 | 1 | The captain who had discovered the incredible island eventually will explore more. | Who will explore more? |
| 65 | 2 | The captain who had discovered the incredible woman eventually will explore more. | Who will explore more? |
| 65 | 3 | The captain who realized that the island was incredible eventually will explore more. | Who will explore more? |
| 65 | 4 | The captain who realized that the woman was incredible eventually will explore more. | Who will explore more? |
| 66 | 1 | The manager who had lied about the cheap car in the end will resign before the summer. | Who will resign? |
| 66 | 2 | The manager who had lied about the cheap associate in the end will resign before the summer. | Who will resign? |
| 66 | 3 | The manager who admitted that the car was cheap in the end will resign before the summer. | Who will resign? |
| 66 | 4 | The manager who admitted that the associate was cheap in the end will resign before the summer. | Who will resign? |
| 67 | 1 | The bandit who had attacked the frail wagon last night was looking for help. | Who was looking for help? |
| 67 | 2 | The bandit who had attacked the frail woman last night was looking for help. | Who was looking for help? |
| 67 | 3 | The bandit who noticed that the wagon was frail last night was looking for help. | Who was looking for help? |
| 67 | 4 | The bandit who noticed that the woman was frail last night was looking for help. | Who was looking for help? |
| 68 | 1 | The worker who had picked up the lost ticket from the front desk will come to the doctor. | Who will come to the doctor? |
| 68 | 2 | The worker who had picked up the lost child from the front desk will come to the doctor. | Who will come to the doctor? |
| 68 | 3 | The worker who saw that the ticket was lost at the front desk will come to the doctor. | Who will come to the doctor? |
| 68 | 4 | The worker who saw that the child was lost at the front desk will come to the doctor. | Who will come to the doctor? |
| 69 | 1 | The businessman who was complaining about the rude delay this afternoon should call the airline. | Who should call? |
| 69 | 2 | The businessman who was complaining about the rude passenger this afternoon should call the airline. | Who should call? |
| 69 | 3 | The businessman who complained that the delay was rude this afternoon should call the airline. | Who should call? |
| 69 | 4 | The businessman who complained that the passenger was rude this afternoon should call the airline. | Who should call? |
| 70 | 1 | The merchant who had sold the creative artwork in the exhibition was lying about the price. | Who was lying? |
| 70 | 2 | The merchant who had sold to the creative artist in the exhibition was lying about the price. | Who was lying? |
| 70 | 3 | The merchant who said that the artwork was creative in the exhibition was lying about the price. | Who was lying? |
| 70 | 4 | The merchant who said that the artist was creative in the exhibition was lying about the price. | Who was lying? |
| 71 | 1 | The player who was practicing on the new field before the game will score many points. | Who will score many points? |
| 71 | 2 | The player who was practicing with the new team before the game will score many points. | Who will score many points? |
| 71 | 3 | The player who realized that the field was new before the game will score many points. | Who will score many points? |
| 71 | 4 | The player who realized that the team was new before the game will score many points. | Who will score many points? |
| 72 | 1 | The conservationist who had supported the pleasant park this year was speaking at the forum. | Who was speaking? |
| 72 | 2 | The conservationist who had supported the pleasant politician this year was speaking at the forum. | Who was speaking? |
| 72 | 3 | The conservationist who remarked that the park was pleasant this year was speaking at the forum. | Who was speaking? |
| 72 | 4 | The conservationist who remarked that the politician was pleasant this year was speaking at the forum. | Who was speaking? |
| 73 | 1 | The editor who had interviewed for the prestigious position yesterday was receiving a reward. | Who was rewarded? |
| 73 | 2 | The editor who had interviewed the prestigious citizen yesterday was receiving a reward. | Who was rewarded? |
| 73 | 3 | The editor who recognized that the position was prestigious yesterday was receiving a reward. | Who was rewarded? |
| 73 | 4 | The editor who recognized that the citizen was prestigious yesterday was receiving a reward. | Who was rewarded? |
| 74 | 1 | The child who was inspired by the creative assignment this summer was thankful for the attention. | Who was thankful? |
| 74 | 2 | The child who was inspired by the creative teacher this summer was thankful for the attention. | Who was thankful? |
| 74 | 3 | The child who said that the assignment was creative this summer was thankful for the attention. | Who was thankful? |
| 74 | 4 | The child who said that the teacher was creative this summer was thankful for the attention. | Who was thankful? |
| 75 | 1 | The boy who had played the interesting game last week had shared the cookies. | Who had shared the cookies? |
| 75 | 2 | The boy who had played with the interesting visitor last week had shared the cookies. | Who had shared the cookies? |
| 75 | 3 | The boy who mumbled that the game was interesting last week had shared the cookies. | Who had shared the cookies? |
| 75 | 4 | The boy who mumbled that the visitor was interesting last week had shared the cookies. | Who had shared the cookies? |
| 76 | 1 | The specialist who has worked with the terminal disease in the hospital will discuss all the options. | Who will discuss all the options? |
| 76 | 2 | The specialist who has worked with the terminal patient in the hospital will discuss all the options. | Who will discuss all the options? |
| 76 | 3 | The specialist who had thinks that the disease is terminal in the hospital will discuss all the options. | Who will discuss all the options? |
| 76 | 4 | The specialist who had thinks that the patient is terminal in the hospital will discuss all the options. | Who will discuss all the options? |
| 77 | 1 | The monster who had captured the precious sword in the battle will regret the action. | Who will regret the action? |
| 77 | 2 | The monster who had captured the precious queen in the battle will regret the action. | Who will regret the action? |
| 77 | 3 | The monster who realized that the sword was precious in the battle will regret the action. | Who will regret the action? |
| 77 | 4 | The monster who realized that the queen was precious in the battle will regret the action. | Who will regret the action? |
| 78 | 1 | The mailman who was avoiding the angry dog last time will knock on the door. | Who will knock? |
| 78 | 2 | The mailman who was avoiding the angry woman last time will knock on the door. | Who will knock? |
| 78 | 3 | The mailman who knew that the dog was angry last time will knock on the door. | Who will knock? |
| 78 | 4 | The mailman who knew that the woman was angry last time will knock on the door. | Who will knock? |
| 79 | 1 | The critic who had liked the interesting painting for years will buy something. | Who will buy something? |
| 79 | 2 | The critic who had liked the interesting painter for years will buy something. | Who will buy something? |
| 79 | 3 | The critic who had thought that the painting was interesting for years will buy something. | Who will buy something? |
| 79 | 4 | The critic who had thought that the painter was interesting for years will buy something. | Who will buy something? |
| 80 | 1 | The cashier who had screamed about the dangerous fire in the lobby was looking for the exit | Who was looking for the exit? |
| 80 | 2 | The cashier who had screamed about the dangerous robber in the lobby was looking for the exit | Who was looking for the exit? |
| 80 | 3 | The cashier who screamed that the fire was dangerous in the lobby was looking for the exit | Who was looking for the exit? |
| 80 | 4 | The cashier who screamed that the robber was dangerous in the lobby was looking for the exit | Who was looking for the exit? |

# Appendix B

Results of the linear mixed-effects models with single predictors on the reading time data. For all single predictors (other than vocabulary), vocabulary was included as a control variable.

|  | **Self-paced reading** | | | | | | **Comprehension question** | | | | | | |
| --- | --- | --- | --- | --- | --- | --- | --- | --- | --- | --- | --- | --- | --- |
| **Coefficients** | **Critical region** | | | **Spillover region** | | | **Question region** | | | **Error rate** | | | |
|  | **Estimate** | **S.E.** | ***t*-value** | **Estimate** | **S.E.** | ***t*-value** | **Estimate** | **S.E.** | ***t*-value** | **Estimate** | **S.E.** | ***z*-value** | **p** |
| ***Vocabulary*** |  |  |  |  |  |  |  |  |  |  |  |  |  |
| (Intercept) | 2.82808 | 0.02090 | 135.30 | 2.86150 | 0.01755 | 163.04 | 3.07341 | 0.01653 | 185.90 | -1.68973 | 0.41317 | 4.09 | 0.000 |
| Semantic | 0.00520 | 0.00267 | 1.95 | 0.00660 | 0.00257 | 2.57 | 0.01928 | 0.00203 | 9.48 | 0.27624 | 0.04889 | -5.65 | 0.000 |
| Syntactic | -0.00008 | 0.00287 | -0.03 | 0.00490 | 0.00245 | 2.00 | 0.00433 | 0.00176 | 2.47 | 0.13068 | 0.04732 | -2.76 | 0.006 |
| Length | 0.00723 | 0.00138 | 5.24 | 0.00618 | 0.00081 | 7.64 | 0.00133 | 0.00067 | 1.98 | -0.03403 | 0.01870 | 1.82 | 0.069 |
| Vocabulary | -0.00650 | 0.00242 | -2.68 | -0.00781 | 0.00247 | -3.16 | -0.00357 | 0.00134 | -2.67 | -0.03405 | 0.01101 | 3.09 | 0.002 |
| Semantic × Syntactic | 0.00178 | 0.00255 | 0.70 | 0.00114 | 0.00223 | 0.51 | 0.00257 | 0.00176 | 1.46 | -0.00463 | 0.04595 | 0.10 | 0.920 |
| Semantic × Vocabulary | 0.00009 | 0.00037 | 0.25 | 0.00015 | 0.00037 | 0.40 | -0.00054 | 0.00033 | -1.65 | 0.01114 | 0.00624 | -1.79 | 0.074 |
| Syntactic × Vocabulary | 0.00024 | 0.00037 | 0.65 | 0.00029 | 0.00036 | 0.82 | 0.00026 | 0.00029 | 0.88 | 0.00827 | 0.00651 | -1.27 | 0.204 |
| ***Category probe*** |  |  |  |  |  |  |  |  |  |  |  |  |  |
| (Intercept) | 2.82800 | 0.02068 | 136.74 | 2.86164 | 0.01744 | 164.12 | 3.07334 | 0.01654 | 185.76 | -1.69545 | 0.41309 | -4.10 | 0.000 |
| Semantic | 0.00524 | 0.00268 | 1.96 | 0.00661 | 0.00258 | 2.57 | 0.01933 | 0.00202 | 9.58 | 0.27717 | 0.04907 | 5.65 | 0.000 |
| Syntactic | -0.00009 | 0.00287 | -0.03 | 0.00492 | 0.00246 | 2.00 | 0.00433 | 0.00176 | 2.46 | 0.13089 | 0.04771 | 2.74 | 0.006 |
| Category | 0.55290 | 0.18040 | 3.06 | 0.34621 | 0.18873 | 1.83 | -0.03859 | 0.10335 | -0.37 | -2.48251 | 0.81920 | -3.03 | 0.002 |
| Vocabulary | -0.00796 | 0.00239 | -3.33 | -0.00875 | 0.00250 | -3.50 | -0.00347 | 0.00137 | -2.54 | -0.02702 | 0.01077 | -2.51 | 0.012 |
| Length | 0.00725 | 0.00138 | 5.27 | 0.00617 | 0.00081 | 7.63 | 0.00133 | 0.00067 | 1.99 | -0.03382 | 0.01871 | -1.81 | 0.071 |
| Semantic × Syntactic | 0.00179 | 0.00255 | 0.70 | 0.00114 | 0.00223 | 0.51 | 0.00255 | 0.00177 | 1.44 | -0.00230 | 0.04624 | -0.05 | 0.960 |
| Semantic × Category | -0.02621 | 0.02867 | -0.91 | -0.00895 | 0.02884 | -0.31 | -0.06306 | 0.02483 | -2.54* | -0.27288 | 0.49294 | -0.55 | 0.580 |
| Syntactic × Category | 0.01888 | 0.02821 | 0.67 | -0.01977 | 0.02736 | -0.72 | -0.00679 | 0.02251 | -0.30 | 0.41798 | 0.51576 | 0.81 | 0.418 |
| Semantic × Vocabulary | 0.00017 | 0.00038 | 0.43 | -0.00013 | 0.00038 | -0.33 | -0.00039 | 0.00033 | -1.18 | 0.01203 | 0.00641 | 1.88 | 0.060 |
| Syntactic × Vocabulary | 0.00018 | 0.00038 | 0.49 | -0.00024 | 0.00037 | -0.64 | 0.00028 | 0.00030 | 0.93 | 0.00686 | 0.00681 | 1.01 | 0.314 |
| ***WM composite*** |  |  |  |  |  |  |  |  |  |  |  |  |  |
| (Intercept) | 2.82900 | 0.02099 | 134.77 | 2.86200 | 0.01750 | 163.54 | 3.07344 | 0.01654 | 185.87 | -1.69745 | 0.41297 | -4.11 | 0.000 |
| Semantic | 0.00517 | 0.00267 | 1.93 | 0.00658 | 0.00257 | 2.56 | 0.01929 | 0.00203 | 9.49 | 0.27599 | 0.04878 | 5.66 | 0.000 |
| Syntactic | -0.00003 | 0.00286 | -0.01 | 0.00496 | 0.00245 | 2.02 | 0.00433 | 0.00176 | 2.47 | 0.13055 | 0.04733 | 2.76 | 0.006 |
| WM composite | -0.00500 | 0.00808 | -0.62 | -0.01013 | 0.00821 | -1.23 | -0.00513 | 0.00443 | -1.16 | -0.09424 | 0.03554 | -2.65 | 0.008 |
| Vocabulary | -0.00597 | 0.00258 | -2.32 | -0.00680 | 0.00262 | -2.60 | -0.00301 | 0.00141 | -2.13 | -0.02412 | 0.01130 | -2.14 | 0.033 |
| Length | 0.00719 | 0.00139 | 5.18 | 0.00617 | 0.00081 | 7.63 | 0.00133 | 0.00067 | 1.98 | -0.03374 | 0.01870 | -1.80 | 0.071 |
| Semantic × Syntactic | 0.00180 | 0.00255 | 0.70 | 0.00118 | 0.00222 | 0.53 | 0.00257 | 0.00176 | 1.46 | -0.00230 | 0.04610 | -0.05 | 0.960 |
| Semantic × WM comp. | 0.00156 | 0.00124 | 1.25 | 0.00067 | 0.00126 | 0.53 | -0.00094 | 0.00111 | -0.84 | -0.03844 | 0.02075 | -1.85 | 0.064 |
| Syntactic × WM comp. | -0.00202 | 0.00121 | -1.66 | -0.00234 | 0.00118 | -1.99 | -0.00036 | 0.00096 | -0.38 | 0.00097 | 0.02142 | 0.05 | 0.964 |
| Semantic × Vocabulary | -0.00008 | 0.00040 | -0.20 | -0.00022 | 0.00040 | -0.56 | -0.00044 | 0.00035 | -1.25 | 0.01514 | 0.00656 | 2.31 | 0.021 |
| Syntactic × Vocabulary | 0.00046 | 0.00039 | 1.19 | -0.00004 | 0.00038 | -0.12 | 0.00030 | 0.00031 | 0.95 | 0.00812 | 0.00692 | 1.17 | 0.241 |
| ***Reading span*** |  |  |  |  |  |  |  |  |  |  |  |  |  |
| (Intercept) | 2.82900 | 0.02097 | 134.89 | 2.86200 | 0.01750 | 163.49 | 3.07345 | 0.01655 | 185.74 | -1.69679 | 0.41296 | -4.11 | 0.000 |
| Semantic | 0.00520 | 0.00269 | 1.94 | 0.00660 | 0.00258 | 2.56 | 0.01930 | 0.00202 | 9.54 | 0.27690 | 0.04872 | 5.68 | 0.000 |
| Syntactic | -0.00004 | 0.00285 | -0.01 | 0.00497 | 0.00244 | 2.03 | 0.00434 | 0.00175 | 2.48 | 0.13105 | 0.04729 | 2.77 | 0.006 |
| Reading | -0.00039 | 0.00138 | -0.28 | -0.00173 | 0.00140 | -1.23 | -0.00078 | 0.00076 | -1.02 | -0.01676 | 0.00604 | -2.77 | 0.006 |
| Vocabulary | -0.00624 | 0.00260 | -2.40 | -0.00670 | 0.00264 | -2.54 | -0.00304 | 0.00143 | -2.13 | -0.02279 | 0.01135 | -2.01 | 0.045 |
| Length | 0.00719 | 0.00138 | 5.20 | 0.00617 | 0.00081 | 7.63 | 0.00133 | 0.00067 | 1.98 | -0.03372 | 0.01870 | -1.80 | 0.071 |
| Semantic × Syntactic | 0.00180 | 0.00255 | 0.70 | 0.00118 | 0.00223 | 0.53 | 0.00257 | 0.00176 | 1.46 | 0.00079 | 0.04610 | 0.02 | 0.986 |
| Semantic × Reading | 0.00014 | 0.00021 | 0.68 | 0.00004 | 0.00021 | 0.20 | -0.00028 | 0.00019 | -1.51 | -0.00642 | 0.00354 | -1.81 | 0.070 |
| Syntactic × Reading | -0.00040 | 0.00020 | -1.97 | -0.00042 | 0.00020 | **-2.10*** | -0.00014 | 0.00016 | -0.86 | -0.00014 | 0.00370 | -0.04 | 0.969 |
| Semantic × Vocabulary | -0.00001 | 0.00040 | -0.02 | -0.00018 | 0.00040 | -0.44 | -0.00035 | 0.00035 | -0.99 | 0.01554 | 0.00663 | 2.35 | 0.019 |
| Syntactic × Vocabulary | 0.00051 | 0.00039 | 1.33 | -0.00001 | 0.00038 | -0.02 | 0.00036 | 0.00031 | 1.13 | 0.00818 | 0.00704 | 1.16 | 0.245 |
| ***Operation span*** |  |  |  |  |  |  |  |  |  |  |  |  |  |
| (Intercept) | 2.82800 | 0.02098 | 134.83 | 2.86157 | 0.01754 | 163.15 | 3.07300 | 0.01653 | 185.91 | -1.69488 | 0.41303 | -4.10 | 0.000 |
| Semantic | 0.00516 | 0.00266 | 1.94 | 0.00658 | 0.00257 | 2.56 | 0.01928 | 0.00204 | 9.45 | 0.27548 | 0.04887 | 5.64 | 0.000 |
| Syntactic | -0.00004 | 0.00287 | -0.02 | 0.00493 | 0.00246 | 2.00 | 0.00433 | 0.00176 | 2.46 | 0.13033 | 0.04736 | 2.75 | 0.006 |
| Operation | -0.00127 | 0.00163 | -0.78 | -0.00150 | 0.00166 | -0.90 | -0.00088 | 0.00090 | -0.98 | -0.01377 | 0.00732 | -1.88 | 0.060 |
| Vocabulary | -0.00606 | 0.00249 | -2.43 | -0.00733 | 0.00254 | -2.88 | -0.00325 | 0.00137 | -2.37 | -0.02931 | 0.01114 | -2.63 | 0.009 |
| Length | 0.00720 | 0.00139 | 5.20 | 0.00617 | 0.00081 | 7.64 | 0.00133 | 0.00067 | 1.98 | -0.03388 | 0.01870 | -1.81 | 0.070 |
| Semantic × Syntactic | 0.00179 | 0.00255 | 0.70 | 0.00116 | 0.00222 | 0.52 | 0.00256 | 0.00176 | 1.45 | -0.00514 | 0.04607 | -0.11 | 0.911 |
| Semantic × Operation | 0.00035 | 0.00025 | 1.43 | 0.00018 | 0.00025 | 0.71 | 0.00000 | 0.00022 | 0.01 | -0.00629 | 0.00426 | -1.48 | 0.140 |
| Syntactic × Operation | -0.00022 | 0.00025 | -0.91 | -0.00032 | 0.00024 | -1.34 | 0.00003 | 0.00019 | 0.18 | 0.00071 | 0.00434 | 0.17 | 0.869 |
| Semantic × Vocabulary | -0.00004 | 0.00038 | -0.10 | -0.00021 | 0.00039 | -0.55 | -0.00054 | 0.00034 | -1.60 | 0.01319 | 0.00639 | 2.07 | 0.039 |
| Syntactic × Vocabulary | 0.00032 | 0.00038 | 0.85 | -0.00018 | 0.00037 | -0.50 | 0.00024 | 0.00030 | 0.80 | 0.00808 | 0.00667 | 1.21 | 0.226 |
| ***Digit span*** |  |  |  |  |  |  |  |  |  |  |  |  |  |
| (Intercept) | 2.82800 | 0.02094 | 135.08 | 2.86137 | 0.01757 | 162.81 | 3.07403 | 0.01653 | 185.94 | -1.69321 | 0.41328 | -4.10 | 0.000 |
| Semantic | 0.00521 | 0.00268 | 1.94 | 0.00660 | 0.00257 | 2.56 | 0.01928 | 0.00203 | 9.49 | 0.27712 | 0.04895 | 5.66 | 0.000 |
| Syntactic | -0.00008 | 0.00287 | -0.03 | 0.00489 | 0.00245 | 2.00 | 0.00434 | 0.00177 | 2.46 | 0.13156 | 0.04727 | 2.78 | 0.005 |
| Digit | -0.03731 | 0.09806 | -0.38 | -0.08971 | 0.09983 | -0.90 | -0.09600 | 0.05334 | -1.80 | -0.33122 | 0.45391 | -0.73 | 0.466 |
| Vocabulary | -0.00635 | 0.00247 | -2.57 | -0.00743 | 0.00251 | -2.96 | -0.00321 | 0.00134 | -2.40 | -0.03265 | 0.01116 | -2.93 | 0.003 |
| Length | 0.00723 | 0.00138 | 5.25 | 0.00619 | 0.00081 | 7.65 | 0.00130 | 0.00067 | 1.93 | -0.03392 | 0.01870 | -1.81 | 0.070 |
| Semantic × Syntactic | 0.00178 | 0.00255 | 0.70 | 0.00115 | 0.00223 | 0.52 | 0.00254 | 0.00178 | 1.42 | -0.00533 | 0.04610 | -0.12 | 0.908 |
| Semantic × Digit | -0.00197 | 0.01507 | -0.13 | -0.00687 | 0.01513 | -0.45 | -0.02394 | 0.01325 | -1.81 | 0.17770 | 0.27063 | 0.66 | 0.511 |
| Syntactic × Digit | 0.00688 | 0.01478 | 0.47 | 0.02301 | 0.01421 | 1.62 | -0.02045 | 0.01180 | -1.73 | 0.24655 | 0.27693 | 0.89 | 0.373 |
| Semantic × Vocabulary | 0.00010 | 0.00038 | 0.27 | -0.00012 | 0.00038 | -0.31 | -0.00045 | 0.00033 | -1.35 | 0.01033 | 0.00635 | 1.63 | 0.104 |
| Syntactic × Vocabulary | 0.00020 | 0.00037 | 0.55 | -0.00040 | 0.00036 | -1.12 | 0.00035 | 0.00030 | 1.18 | 0.00699 | 0.00664 | 1.05 | 0.293 |
| ***Stroop*** |  |  |  |  |  |  |  |  |  |  |  |  |  |
| (Intercept) | 2.82800 | 0.02096 | 134.95 | 2.86100 | 0.01751 | 163.45 | 3.07300 | 0.01654 | 185.87 | -1.69000 | 0.41330 | -4.09 | 0.000 |
| Semantic | 0.00519 | 0.00267 | 1.94 | 0.00661 | 0.00257 | 2.57 | 0.01928 | 0.00204 | 9.45 | 0.27700 | 0.04895 | 5.66 | 0.000 |
| Syntactic | -0.00008 | 0.00287 | -0.03 | 0.00491 | 0.00246 | 2.00 | 0.00434 | 0.00176 | 2.47 | 0.13010 | 0.04746 | 2.74 | 0.006 |
| Stroop | 0.00006 | 0.00023 | 0.27 | 0.00031 | 0.00023 | 1.32 | 0.00011 | 0.00013 | 0.89 | -0.03406 | 0.01870 | -1.82 | 0.069 |
| Vocabulary | -0.00639 | 0.00247 | -2.59 | -0.00728 | 0.00250 | -2.91 | -0.00337 | 0.00136 | -2.49 | 0.00098 | 0.00105 | 0.93 | 0.351 |
| Length | 0.00722 | 0.00138 | 5.23 | 0.00618 | 0.00081 | 7.65 | 0.00133 | 0.00067 | 1.98 | -0.03253 | 0.01112 | -2.93 | 0.003 |
| Semantic × Syntactic | 0.00177 | 0.00256 | 0.69 | 0.00115 | 0.00223 | 0.51 | 0.00257 | 0.00176 | 1.46 | -0.00252 | 0.04592 | -0.06 | 0.956 |
| Semantic × Stroop | -0.00004 | 0.00004 | -1.04 | 0.00003 | 0.00004 | 0.92 | -0.00002 | 0.00003 | -0.67 | -0.00032 | 0.00061 | -0.53 | 0.596 |
| Syntactic × Stroop | -0.000005 | 0.00003 | -0.13 | 0.00000 | 0.00003 | -0.04 | 0.00002 | 0.00003 | 0.83 | -0.00004 | 0.00063 | -0.06 | 0.953 |
| Semantic × Vocabulary | 0.00003 | 0.00038 | 0.09 | -0.00010 | 0.00038 | -0.26 | -0.00058 | 0.00033 | -1.73 | 0.01067 | 0.00631 | 1.69 | 0.091 |
| Syntactic × Vocabulary | 0.00023 | 0.00037 | 0.62 | -0.00030 | 0.00036 | -0.82 | 0.00029 | 0.00030 | 0.99 | 0.00820 | 0.00655 | 1.25 | 0.211 |

# Appendix C

Reliability estimates for all the dependent measurements. Internal reliability was calculated as the split-half correlation adjusted with the Spearman-Brown prophecy formula (Cronbach, 1951).

|  | Sentence reading | | Comprehension question | |
| --- | --- | --- | --- | --- |
|  | RT (Critical) | RT (Spillover) | RT (Question) | Accuracy |
| **Reliability** | .99 | .99 | .96 | .78 |

# Appendix D

Results of mixed-effects model, which included all individual differences measures except operation span. Random intercepts for subjects and items, as well as random slopes for semantic ×syntactic interference manipulations were included.

|  | Comprehension Question | | | | | | |  | Self-paced Reading (ms) | | | | | |
| --- | --- | --- | --- | --- | --- | --- | --- | --- | --- | --- | --- | --- | --- | --- |
|  | **Error rate** | | | | **Speed (RT)** | | |  | **Critical region (RT)** | | | **Spillover region (RT)** | | |
|  | **Coefficient** | **SE** | ***z*-score/ *p*-value** | | **Coefficient** | **SE** | ***t-*score** |  | **Coefficient** | **SE** | ***t-*score** | **Coefficient** | **SE** | ***t-*score** |
| Intercept | -1.70400 | 0.41310 | **-4.12*** | 0.000 | 3.07400 | 0.01657 | **185.52*** |  | 2.82800 | 0.02078 | 136.12 | 2.86200 | 0.01733 | **165.16*** |
| Length | -0.03351 | 0.01872 | -1.79 | 0.073 | 0.00130 | 0.00067 | 1.94 |  | 0.00720 | 0.00138 | 5.21 | 0.00618 | 0.00081 | **7.64*** |
| Semantic interference | 0.27960 | 0.04892 | **5.71*** | 0.000 | 0.01933 | 0.00202 | **9.57*** |  | 0.00523 | 0.00269 | 1.94 | 0.00662 | 0.00259 | **2.55*** |
| Syntactic interference | 0.13190 | 0.04760 | **2.77*** | 0.006 | 0.00434 | 0.00177 | **2.45*** |  | -0.00007 | 0.00286 | -0.02 | 0.00496 | 0.00244 | **2.03*** |
| Semantic ×Syntactic | 0.00136 | 0.04639 | 0.03 | 0.977 | 0.00253 | 0.00178 | 1.42 |  | 0.00182 | 0.00255 | 0.71 | 0.00119 | 0.00222 | 0.53 |
| *IDs* |  |  |  |  |  |  |  |  |  |  |  |  |  |  |
| Category probe | -2.03900 | 0.83870 | **-2.43*** | 0.015 | -0.01034 | 0.10740 | -0.10 |  | 0.60810 | 0.18960 | 3.21 | 0.44100 | 0.19530 | **2.26*** |
| ×Semantic | -0.01315 | 0.51230 | -0.03 | 0.980 | -0.05375 | 0.02585 | **-2.08*** |  | -0.03103 | 0.02993 | -1.04 | -0.01211 | 0.03020 | -0.40 |
| ×Syntactic | 0.44810 | 0.53840 | 0.83 | 0.405 | -0.00110 | 0.02362 | -0.05 |  | 0.03351 | 0.02891 | 1.16 | -0.00629 | 0.02805 | -0.22 |
| Reading span | -0.01192 | 0.00629 | -1.90 | 0.058 | -0.00044 | 0.00082 | -0.54 |  | -0.00141 | 0.00144 | -0.98 | -0.00220 | 0.00148 | -1.48 |
| ×Semantic | -0.00739 | 0.00380 | -1.94 | 0.052 | -0.00015 | 0.00020 | -0.75 |  | 0.00017 | 0.00023 | 0.73 | 0.00012 | 0.00023 | 0.53 |
| ×Syntactic | -0.00151 | 0.00398 | -0.38 | 0.705 | -0.00008 | 0.00018 | -0.45 |  | -0.00051 | 0.00022 | **-2.32*** | -0.00049 | 0.00021 | **-2.31*** |
| Digit span | -0.06163 | 0.43640 | -0.14 | 0.888 | -0.08632 | 0.05489 | -1.57 |  | -0.04038 | 0.09692 | -0.42 | -0.07137 | 0.09976 | -0.72 |
| ×Semantic | 0.23480 | 0.27590 | 0.85 | 0.395 | -0.02123 | 0.01341 | -1.58 |  | -0.00472 | 0.01547 | -0.31 | -0.00604 | 0.01564 | -0.39 |
| ×Syntactic | 0.25060 | 0.28750 | 0.87 | 0.383 | -0.01854 | 0.01211 | -1.53 |  | 0.01078 | 0.01490 | 0.72 | 0.02909 | 0.01444 | **2.01*** |
| Stroop | 0.00061 | 0.00102 | 0.60 | 0.546 | 0.00008 | 0.00013 | 0.59 |  | 0.00001 | 0.00023 | 0.04 | 0.00022 | 0.00024 | 0.94 |
| ×Semantic | -0.00048 | 0.00062 | -0.78 | 0.435 | -0.00003 | 0.00003 | -1.00 |  | -0.00003 | 0.00004 | -0.90 | 0.00003 | 0.00004 | 0.93 |
| ×Syntactic | -0.00001 | 0.00066 | -0.01 | 0.991 | 0.00001 | 0.00003 | 0.49 |  | -0.00002 | 0.00004 | -0.51 | -0.00001 | 0.00003 | -0.28 |
| Vocabulary | -0.01904 | 0.01119 | -1.70 | 0.089 | -0.00279 | 0.00145 | -1.93 |  | -0.00700 | 0.00256 | -2.74 | -0.00691 | 0.00263 | **-2.62*** |
| ×Semantic | 0.01459 | 0.00674 | **2.16*** | 0.030 | -0.00028 | 0.00035 | -0.81 |  | 0.00003 | 0.00041 | 0.07 | -0.00011 | 0.00041 | -0.27 |
| ×Syntactic | 0.00641 | 0.00724 | 0.89 | 0.376 | 0.00043 | 0.00032 | 1.33 |  | 0.00042 | 0.00040 | 1.05 | -0.00009 | 0.00038 | -0.24 |

Note. A coefficient is a significant predictor of RT or accuracy of comprehension question at *p* < .05 with criterion that |t| or |z| >=2.

# Appendix E

Results of mixed-effects model, which included all individual differences measures except reading span. Random intercepts for subjects and items, as well as random slopes for semantic ×syntactic interference manipulations were included.

|  | Comprehension Question | | | | | | |  | Self-paced Reading (ms) | | | | | |
| --- | --- | --- | --- | --- | --- | --- | --- | --- | --- | --- | --- | --- | --- | --- |
|  | **Error rate** | | | | **Speed (RT)** | | |  | **Critical region (RT)** | | | **Spillover region (RT)** | | |
|  | **Coefficient** | **SE** | ***z*-score/ *p*-value** | | **Coefficient** | **SE** | ***t-*score** |  | **Coefficient** | **SE** | ***t-*score** | **Coefficient** | **SE** | ***t-*score** |
| Intercept | -1.70300 | 0.41320 | **-4.12*** | 0.000 | 3.07400 | 0.01656 | **185.69*** |  | 2.82800 | 0.02083 | 135.77 | 2.86200 | 0.01742 | **164.30*** |
| Length | -0.03361 | 0.01872 | -1.80 | 0.072 | 0.00130 | 0.00067 | 1.93 |  | 0.00722 | 0.00139 | 5.20 | 0.00618 | 0.00081 | **7.64*** |
| Semantic interference | 0.27780 | 0.04917 | **5.65*** | 0.000 | 0.01933 | 0.00203 | **9.51*** |  | 0.00519 | 0.00267 | 1.94 | 0.00660 | 0.00258 | **2.56*** |
| Syntactic interference | 0.13040 | 0.04777 | **2.73*** | 0.006 | 0.00434 | 0.00178 | **2.44*** |  | -0.00007 | 0.00288 | -0.02 | 0.00493 | 0.00246 | **2.01*** |
| Semantic ×Syntactic | -0.00289 | 0.04641 | -0.06 | 0.950 | 0.00252 | 0.00179 | 1.41 |  | 0.00181 | 0.00255 | 0.71 | 0.00118 | 0.00222 | 0.53 |
| *IDs* |  |  |  |  |  |  |  |  |  |  |  |  |  |  |
| Category probe | -2.33300 | 0.82450 | **-2.83*** | 0.005 | -0.02194 | 0.10460 | -0.21 |  | 0.58810 | 0.18410 | **3.19*** | 0.38110 | 0.19130 | 1.99 |
| ×Semantic | -0.16890 | 0.50220 | -0.34 | 0.737 | -0.06172 | 0.02522 | **-2.45*** |  | -0.03055 | 0.02885 | -1.06 | -0.01186 | 0.02923 | -0.41 |
| ×Syntactic | 0.39430 | 0.52340 | 0.75 | 0.451 | -0.00701 | 0.02306 | -0.30 |  | 0.02175 | 0.02874 | 0.76 | -0.01661 | 0.02752 | -0.60 |
| Operation span | -0.00945 | 0.00757 | -1.25 | 0.212 | -0.00028 | 0.00097 | -0.29 |  | -0.00188 | 0.00171 | -1.10 | -0.00124 | 0.00177 | -0.70 |
| ×Semantic | -0.00069 | 0.00470 | -0.15 | 0.884 | 0.00019 | 0.00021 | 0.89 |  | -0.00033 | 0.00027 | -1.21 | -0.00047 | 0.00026 | -1.85 |
| ×Syntactic | -0.00807 | 0.00458 | -1.76 | 0.078 | 0.00019 | 0.00024 | 0.79 |  | 0.00039 | 0.00027 | 1.44 | 0.00031 | 0.00028 | 1.13 |
| Digit span | -0.02994 | 0.45160 | -0.07 | 0.947 | -0.08644 | 0.05636 | -1.53 |  | -0.02510 | 0.09931 | -0.25 | -0.07352 | 0.10310 | -0.71 |
| ×Semantic | 0.29020 | 0.28490 | 1.02 | 0.308 | -0.02614 | 0.01388 | -1.88 |  | -0.00965 | 0.01580 | -0.61 | -0.01011 | 0.01606 | -0.63 |
| ×Syntactic | 0.24270 | 0.29310 | 0.83 | 0.408 | -0.02224 | 0.01246 | -1.78 |  | 0.01149 | 0.01566 | 0.73 | 0.03200 | 0.01497 | **2.14*** |
| Stroop | 0.00066 | 0.00103 | 0.64 | 0.525 | 0.00008 | 0.00013 | 0.62 |  | 0.00000 | 0.00023 | -0.02 | 0.00025 | 0.00024 | 1.04 |
| ×Semantic | -0.00050 | 0.00063 | -0.79 | 0.430 | -0.00002 | 0.00003 | -0.69 |  | -0.00003 | 0.00004 | -0.73 | 0.00004 | 0.00004 | 1.06 |
| ×Syntactic | 0.00001 | 0.00066 | 0.02 | 0.984 | 0.00002 | 0.00003 | 0.78 |  | -0.00001 | 0.00004 | -0.33 | -0.00001 | 0.00003 | -0.24 |
| Vocabulary | -0.02301 | 0.01099 | **-2.09*** | 0.036 | -0.00295 | 0.00141 | **-2.09*** |  | -0.00731 | 0.00248 | -2.94 | -0.00770 | 0.00258 | **-2.99*** |
| ×Semantic | 0.01231 | 0.00660 | 1.87 | 0.062 | -0.00040 | 0.00034 | -1.17 |  | 0.00003 | 0.00039 | 0.08 | -0.00011 | 0.00040 | -0.29 |
| ×Syntactic | 0.00600 | 0.00700 | 0.86 | 0.391 | 0.00035 | 0.00032 | 1.12 |  | 0.00023 | 0.00039 | 0.58 | -0.00025 | 0.00038 | -0.65 |

Note. A coefficient is a significant predictor of RT or accuracy of comprehension question at *p* < .05 with criterion that |t| or |z| >=2.

# Appendix F

Comparison of nested models with reading span, operation span, or both.

| Model | Fixed effects |  |  | Comparison | DV | | |
| --- | --- | --- | --- | --- | --- | --- | --- |
|  | Category probe, Stroop,  Digit span,  Vocabulary | Reading span | Operation span | Likelihood Ratio Test | Critical  (RT) | Spillover (RT) | Question (RT) |
| Model 0 | Yes |  |  |  |  |  |  |
| Model 2 | Yes | Yes |  | Model 2 vs. 0 | χ^2^ (3) = 7.42, *p* = .**06** | χ^2^ (3) = 8.21, *p* = **.04*** | χ^2^ (3) = .90, *p* = .83 |
| Model all | Yes | Yes | Yes | Model all vs. 2 | χ^2^ (3) = 2.22, *p* = .53 | χ^2^ (3) = 1.96, *p* = .58 | χ^2^ (3) = 2.91, *p* = .41 |
|  |  |  |  |  |  |  |  |
| Model 3 | Yes |  | Yes | Model 3 vs. 0 | χ^2^ (3) = 5.25, *p* = .15 | χ^2^ (3) = 5.51, *p* = .14 | χ^2^ (3) = 1.45, *p* = .69 |
| Model all | Yes | Yes | Yes | Model all vs. 3 | χ^2^ (3) = 4.40, *p* = .22 | χ^2^ (3) = 4.66, *p* = .20 | χ^2^ (3) = 2.37, *p* = .50 |

# Appendix G

Scatter plots of the significant interactions in mixed-effects analysis with single predictors on residualized log RTs (variance shared with region length was removed) and error rate. Fig. A shows the Syntactic interference × WM span (composite score) interaction in self-paced reading time (ms) in the critical region (“*was complaining*”) and the spillover region (“*about the investivation*”). Fig. B shows the Semantic interference × Category probe, the Semantic interference × Vocabulary, and the Semantic interference × WM interactions in question answering speed and error rate.


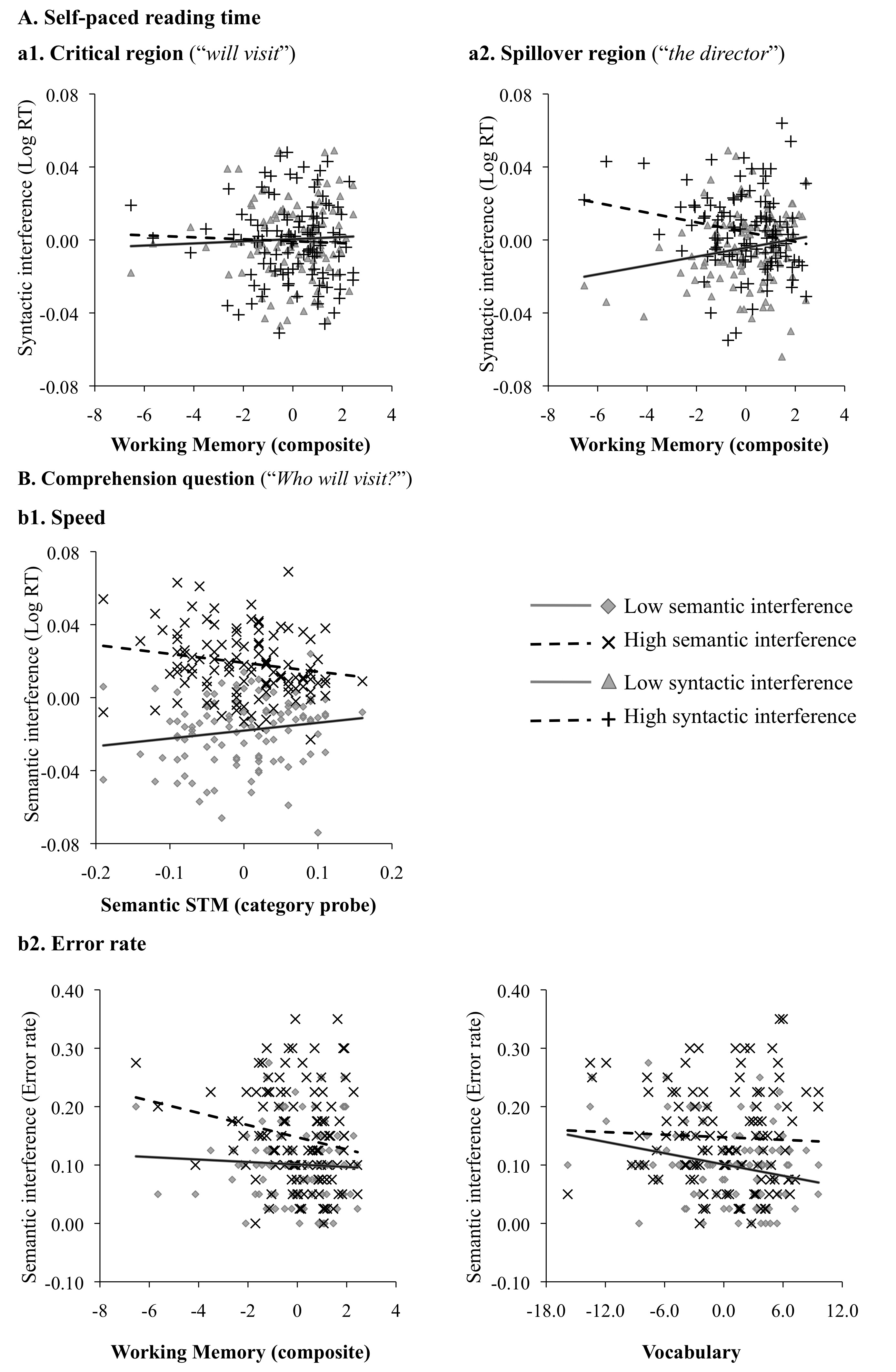

Supplement: Supplementary file 1 [file DataSheet1.DOCX]
